# Connectivity of Edaphic and Endolithic Microbial Niches in Cold Mountain Desert of Eastern Pamir (Tajikistan)

**DOI:** 10.3390/biology10040314

**Published:** 2021-04-09

**Authors:** Nataliia Khomutovska, Asunción de los Ríos, Marcin D. Syczewski, Iwona Jasser

**Affiliations:** 1 Institute of Environmental Biology, Faculty of Biology, Biological and Chemical Research Centre, University of Warsaw, Żwirki i Wigury 101, 02-089 Warsaw, Poland; jasser.iwona@biol.uw.edu.pl; 2 Department of Biogeochemistry and Microbial Ecology, The National Museum of Natural Sciences-CSIC, 28006 Madrid, Spain; arios@mncn.csic.es; 3 Institute of Geochemistry, Mineralogy and Petrology, Faculty of Geology, University of Warsaw, 02-089 Warsaw, Poland; marcinsyczewski@uw.edu.pl

**Keywords:** endoliths, cyanobacteria, biological soil crusts (BSCs), microbial community, Eastern Pamir, cold mountain desert, 16S rRNA gene

## Abstract

**Simple Summary:**

Microbial communities play a significant role in the functioning of the ecosystem in the cold mountain desert of Eastern Pamir, a region that is characterized by extreme environments with limited nutrient and water availability and sparse vegetation. This study aims to investigate the taxonomic composition and structure of bacterial endolithic (rock matrix colonizing) assemblages and soil-inhabiting communities that occur in the Eastern Pamir Mountains, and to compare the composition and structure of both types of communities looking for general patterns and trying to identify the connectivity between these types of microbial assemblages. The taxonomic composition of Pamirian endolithic communities and soil-inhabiting microbial consortia (biocrust communities) exhibit similarities to communities from cold and hot deserts, but they also harbor taxa, which are possibly novel species or which, so far, lack reference sequences in the databases. The endolithic communities occurring in three subregions of Eastern Pamir are characterized by high heterogeneity and cannot be grouped according to the subregion, while the distribution of Cyanobacteria in the soil crusts demonstrates the similarities between the communities within the subregion reflecting regional differences. We also found connectivity between both habitats concerning the occurrence the same taxa (e.g., Cyanobacteria).

**Abstract:**

Microbial communities found in arid environments are commonly represented by biological soil crusts (BSCs) and endolithic assemblages. There is still limited knowledge concerning endoliths and BSCs occurring in the cold mountain desert of Pamir. The aim of the study was to investigate the composition and structure of endolithic bacterial communities in comparison to surrounding BSCs in three subregions of the Eastern Pamir (Tajikistan). The endolithic and BSC communities were studied using culture-independent and culture-dependent techniques. The structure of the endolithic bacterial communities can be characterized as Actinobacteria–Proteobacteria–Bacteroidetes–Chloroflexi–Cyanobacteria, while the BSCs’ can be described as Proteobacteria–Actinobacteria–Bacteroidetes–Cyanobacteria assemblages with low representation of other bacteria. The endolithic cyanobacterial communities were characterized by the high percentage of Chroococcidiopsaceae, Nodosilineaceae, Nostocaceae and Thermosynechococcaceae, while in the BSCs were dominated by Nodosilineaceae, Phormidiaceae and Nostocaceae. The analysis of 16S rRNA genes of the cyanobacterial cultures revealed the presence of possibly novel species of *Chroococcidiopsis*, *Gloeocapsopsis* and *Wilmottia*. Despite the niches’ specificity, which is related to the influence of microenvironment factors on the composition and structure of endolithic communities, our results illustrate the interrelation between the endoliths and the surrounding BSCs in some regions. The structure of cyanobacterial communities from BSC was the only one to demonstrate some subregional differences.

## 1. Introduction

Arid environments constitute a large part of the Earth’s landmass. They are sensitive to climate changes and have a tendency for expansion [1]. Thus, they are considered to be important areas for the monitoring of the influence of global climate change on biodiversity in ecosystems with limited nutrient and water availability [2,3]. The drylands of Central Asia, including the Pamir Mountains, are one of the more poorly studied regions with regards to the diversity and distribution of microbial communities. Eastern Pamir is part of a mountain range, which can be characterized as a cold mountain desert, with sparse vegetation, low nutrient concentration, and low precipitation, and severe climatic conditions [4,5]. Due to its contrasting climatic conditions, complex geology, and varying salinity of the soil, Eastern Pamir is rich in different niches inhabited by microbial communities; studying them provides a good opportunity for studying the microorganisms and the influence of climate change on their development and functioning. The organisms inhabiting such ecosystems are exposed to trophic selective pressure at the local and regional scale [6] and need to be adapted and resistant to niche filtering/specification [7]. Ubiquitous microorganisms are associated with different niches in desert ecosystems, including water reservoirs (planktonic and benthic organisms), desert soil (forming biological soil crusts), rocks (lithobionts) and the air (in the form of aerosols) [2,8,9]. The lithobiontic communities from Asian deserts are still poorly explored concerning both the taxonomic and functional diversity [10]. The results of comparative analysis of lithobiontic communities and biological soil crust systems published so far indicate that these two niches seem to be remarkably isolated [7,11,12]. Both types of microbial communities, endolithic and edaphic ones, are water-dependent but can tolerate desiccation and can recover after a long period of dehydration [13]. While rock architecture and porosity of the lithic substrate shape the endolithic colonization [14,15], edaphic communities are dependent on soil attributes [16,17,18]. Even though endolithic and edaphic microbial communities are often located in proximity to each other, microorganisms of both types of assemblages occupy distinct microhabitats characterized by different microenvironmental conditions, which consequently support the development of divergent communities [12].

Both types of microbial systems are significant for the functioning of desert ecosystems, including their role in basic biochemical processes such as carbon and nitrogen sequestration, nutrients cycling, biological rock weathering and pedogenesis (lithobionts), soil-geomorphic processes and stabilization of soil (soil-inhabiting microorganisms) [2,19,20,21,22]. Some phyla of Bacteria, such as Cyanobacteria, are considered key players in the biogeochemical processes [11,21] due to their metabolic abilities [11,23,24,25] and their soil-stabilizing potential [26]. For this reason, special attention has been paid to this taxonomic group. Cyanobacteria are considered pioneer microorganisms; they enrich barren soil in nitrogen and carbon in the forms available for plants. They also release vitamins, phytohormones and phosphorus improving the fertility of the soil [27]. The results of the experiments using different species of nitrogen-fixing and non-N-fixing Cyanobacteria as an inoculum and different types of soil demonstrated the positive effect of this taxonomic group of Bacteria on the soil stabilization and stability of the concentration of organic N and C [27].

There are only a few studies comparing the composition and structure of both niches in the desert ecosystems of the Earth and they concerned communities from Antarctica [12,20] and those from Colorado Plateau [7]. To our knowledge, there has been no study, until now, concerning bacterial diversity of biological soil crusts-inhabiting taxa occurring in the cold desert of Eastern Pamir. There have only been only a few studies, our preceding research, done on the diversity of endolithic communities from this region [10,28].

The previously described differences in climatic conditions prevailing across the high mountains plateau, and divergent hydrology and geomorphology of Eastern Pamir [4,5,29,30], allowed us to divide the study area into three subregions. They are: Bulunkul in the south, with the wetter and coldest conditions, compared to the Rangkul subregion, which is drier, but a little warmer and is located north-east from Bulunkul, and the third, Karakul located north from Bulunkul and Rangkul and characterized by its dry and cold climatic conditions [4,5]. Endolithic colonization has been previously analyzed only in selected types of rocks (e.g., granites, quartzite, limestones and gneiss) and geographical locations [10,28].

Taking into account the specific climatic and geological conditions and geographical differences of localities from Eastern Pamir, analyzed within the present study, we hypothesize that: (i) there are regional differences in the distribution of taxonomically different groups of endolithic bacteria occurring in the Eastern Pamir compared to those described from other cold and hot deserts; (ii) geomorphology of region and climate have a great influence on diversity and structure of the endolithic communities occurring in different subregions of Eastern Pamir and (iii) there is connectivity between communities occurring in the endolithic and edaphic habitats.

Hence, the aims of the present study were (1) to investigate the taxonomic composition and structure of endolithic bacterial community, which occur in different types of rocks and along geomorphologically different zones of the Eastern Pamir Mountains; (2) to characterize the diversity and structure of biological soil crust (BSC) systems in these subregions; (3) to estimate the contribution of cyanobacteria at both types of communities (endolithic and edaphic) and (4) to compare the composition and structure of both types of communities looking for general patterns and trying to identify the connectivity between these types of microbial assemblages.

## 2. Materials and Methods

### 2.1. Field Area, Sampling and Study Design

The field area of the study comprises Eastern Pamir, which is located in the eastern part of the Gorno-Badakhshan province of Tajikistan. The majority of Eastern Pamir is a barren area with sparse vegetation, where the biodiversity spots are concentrated around the lakes of Pamir, hot springs and, sometimes drying out, rivers. The top of the mountains, where environmental conditions are even harsher than at lower altitudes, are characterized by rocky surfaces and widely distributed endolithic colonization. The geology of Eastern Pamir is very complex and based on the generalized classification, the area can be divided into three main zones: the northern part/zone dominated by volcanic rocks; the central part dominated by sedimentary rocks, and the southern zone mainly represented by metamorphic bedrock [29,30]. For each geological zone the occurrence of rocks different in origin and age from the dominating in the zone was also noted [30].

The study took place in three subregions of Eastern Pamir, which fall within the main geological zones of the Pamir Mountains, near lakes; Karakul—the northern zone, Rangkul—central zone and Bulunkul—the most southern zone [29,30] The subregions were named according to the names of meteorological stations from which were collected data concerning the climate (Karakul and Bulunkul) or according to the name of the largest lake in the area (as in case of Rangkul). These subregions are characterized based on their geographic location, hydrology, climatic conditions and geology. The study area can be characterized as a mountainous plateau at 3700–4000 m above sea level (m a.s.l.); the subregions are divided by high mountain ranges, which, apart from distance in kilometers, isolate them from each other. The lakes belong to different watersheds, which are not connected to each other; Bulunkul to Yashikul watershed [5,31] while Karakul and Rangkul forming their own small and enclosed catchment areas with no outflows. The straight line distance between Bulunkul and Rangkul is about 135 km, from Rangkul to Karakul is approximately 80 km, and the distance from Bulunkul to Karakul is 160 km, but the distance along the valleys and mountain passes connecting the subregions is much larger. 

**Figure 1:**
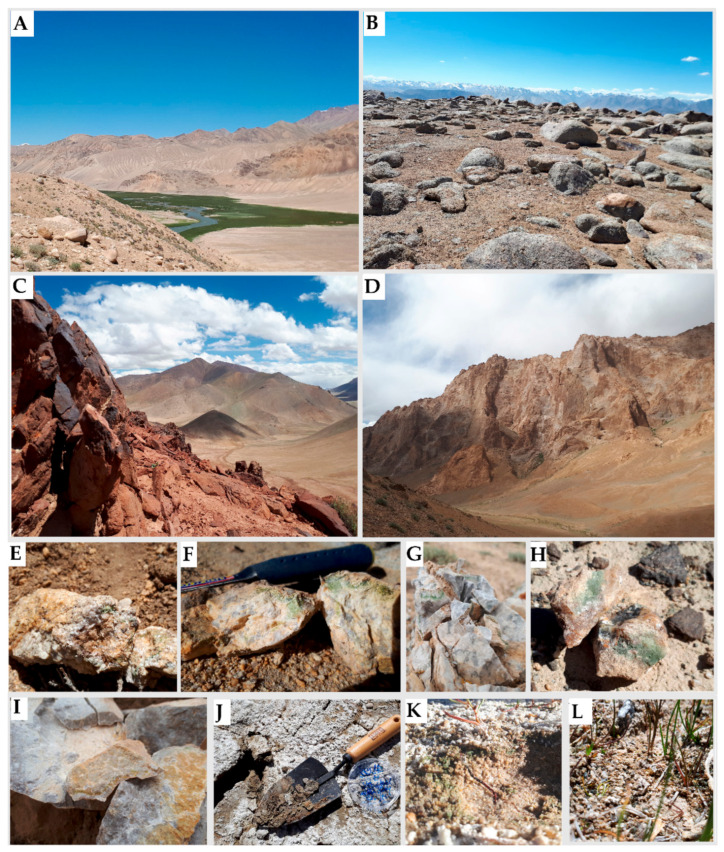
Field sites and investigated types of samples. (**A**) The field area near Bulunkul Lake. (**B**) Sampling site near Karakul. (**C**,**D**) The view from the mountainside near Rangkul. (**E**,**F**) Pegmatites, (**G**) limestone, (**H**) quartzite and (**I**) calcites with endolithic colonization and biological soil crusts collected from (**J**) Rangkul, (**K**) Bulunkul and (**L**) Karakul.

Forty-three of the studied samples of endoliths have been collected in July 2017, which according to the recently developed study [32], shall be enough to investigate the diversity of lithobionts. The studied rocks have been classified as granites (11), limestones (8), quartzites (8), calcites (5), conglomerates (3), pegmatites (3), amphibolites (2), diorite (1), kaolinite (1) and regolith (1) (Supplementary Table S1A; Figure 1). The main sample set includes granites, quartzites and limestones; the other samples were used as a reference for comparison due to their less frequent occurrence. The samples IDs contain the first letter according to the type of rock and the last letter referring to the area from which the sample has been collected.

The samples with endolithic colonization have been split up into four subsamples intended for (1) for molecular analysis, (2) isolation of cyanobacteria, (3) SEM microscopy and (4) the back-up sample. The BSCs were sampled from areas surrounding the rocks from which the endolithic specimens came. In some sampling areas, the diversity of the rock types was high, while in the others the soil seemed to be more heterogeneous and the representations of rocks within the sampling area were less diverse. Thus, the sampling was aimed to screen different types of microhabitats colonized by microorganisms as accurately as it was possible. The diversity and distribution of cyanobacteria in the endolithic communities were compared with these parameters of the soil-inhabiting communities. The sampling of endoliths and BSC was performed concurrently.

Twenty-seven samples of the BSCs were collected to characterize the diversity, composition and structure and the distribution of bacteria in this microhabitat and to compare with the endolithic microhabitats. The subsampling of the BSCs was performed in a similar way as in the case of endoliths, but instead of SEM examination, the samples of biocrusts were tested through chemical analysis of the soil, which is described in the next section.

### 2.2. Chemical Analysis of the Soil

The chemical analysis of the soil was performed in the Laboratory of Biogeochemistry and Environmental Conservation, at the University of Warsaw. Dried samples of soil crusts have been ground and sieved with a 1 mm mesh sieve. The pH of the soil and the mineral parts content was investigated based on samples’ ignition at 550 °C for 90 min. Total C and total N (CHNS NA2500 Thermoquest elemental analyzer, CE Instruments Ltd., Wigan, UK). Total K, Na, Ca, Mg and Fe contents in soil have been estimated after the digestion in nitric/hydrofluoric acids (10:1) with Speedwave 4 microwave mineralizer (Berghof Products + Instruments GmbH, Eningen, Germany) [33,34].

### 2.3. Electron Microscopy Analysis with Energy Dispersive Spectroscopy (SEM-EDS) and Transmission Electron Microscopy (TEM)

The environmental samples of endoliths were examined under the FE-SIGMA VP microscope (Carl Zeiss Microscopy GmbH, Jena, Germany) with an energy-dispersive (EDS) detector (Quantax XFlash 6|10, Bruker NanoGmbH, Berlin, Germany), based on the procedure described in our previous work [28]. Small fragments of rocks were placed on the aluminum mount with carbon conductive tape. Then, the samples were coated in a 20 nm layer of carbon by a vacuum coater (Quorum 150T ES, Laughton, East Sussex, UK). The carbon tape bridges were made for each sample, to avoid excessive accumulation of charge. The analysis was done with a 60 μm aperture and 15 keV acceleration voltage. The beam intensity was 2.5 nA. The working distance depended on the sample height and it was chosen to reach about 8 mm.

The cross-sections of rock samples were prepared to assess the available pore space (porosity) of the rocks. The porosity was analyzed based on two parameters: the mean grain size (MGS), and the mean grain area (MGA). Up to 10 images were done, based on the cross-section per sample using SEM. Next, the images were binarized and analyzed using Gwyddion software according to the user guidelines instructions [35].

The ultrastructure of cyanobacterial cells was analyzed using transmission electron microscopy (TEM). The samples were prepared following the protocol described by de los Ríos and Ascaso [36]. The cyanobacteria cells were initially fixed in glutaraldehyde (2.5% *v*/*v* in phosphate buffer), then postfixed in osmium tetroxide (1% *w*/*v* in phosphate buffer) and finally dehydrated in a graded ethanol series before embedding in Spurr’s resin. Ultrathin sections (70 nm) were post-stained with lead citrate and observed in a JEOL JEM-1011 transmission electron microscope.

### 2.4. Isolation, Cultivation and Morphological Analysis of Cyanobacteria

Fragments of rocks with endolithic colonization have been isolated and inserted into Petri dishes with solid (1.5–2% agar) and liquid WC or BG11 medium [37,38]. The growth conditions in the plant growth chamber were as follows: 12 h/12 h light/dark conditions with cool white light, at 16/18 °C [10].

Selected cultures were restreaked, then purified by washing in ultraclean water and maintained in 50- or 100-mL Erlenmeyer flasks containing BG11 or WC. Next, four strains were prepared for TEM analysis and the sequencing of two regions from the cyanobacterial genome, a fragment of the 16S ribosomal RNA gene and the 16S-23S internal transcribed spacer (ITS).

### 2.5. DNA Extraction, PCR and Illumina MiSeq Sequencing

The sample preparation for DNA extraction has been described previously [10,28]. Extraction was performed by applying the Soil DNA Purification Kit (GeneMATRIX, EURx Ltd.; Gdańsk, Poland) and E.Z.N.A.^®^Soil DNA Kit (Omega Bio-tek, Norcross, GA, USA) following the manufacturer’s protocol. The extraction was repeated three to five times per sample until satisfactory DNA quality has been reached. The amplification of a targeted fragment was conducted using a primer pair of 341 f/785 r [39] and HotStarTaq DNA Polymerase (Qiagen, Hilden, Germany). The amplification procedure of the V3-V4 hypervariable region of the 16S rRNA gene and reaction mix preparation have been described in our previous study [10]. The sequencing was carried out commercially in “Bionanopark’’ (Łódź, Poland) applying the Illumina MiSeq System (2 × 300 bp paired-end) based on the v3 MiSeq Reagent Kit. The depth of the sequencing was 110,000.0 reads/sample. The obtained sequencing results are presented in Supplementary Figure S2. The amplification of a fragment of 16S rRNA gene and the 16S-23S ITS region of selected cyanobacterial strains was conducted in the Department of Biogeochemistry and Microbial Ecology (Madrid, Spain) using the primer pair CYA359F [40] and 373R [41]. The PCR reaction mix contains 3 mL of DNA template, 1.25 µM of each primer and 19.5 mL nuclease-free water. The PCR conditions were as follows: 15 min of denaturation at 95 °C, followed by 35 cycles of 94 °C for 30 s, 50 °C for 90 s, 72 °C for 60 s and final elongation at 60 °C for 30 min. The sequencing was performed commercially by Macrogen Europe (Madrid, Spain). 

### 2.6. Bioinformatic Analysis and Statistics

Paired-end reads have been processed using version 2020.11 of the QIIME2 environment [42]. The “demux” plugin was used to view the quality of sequences. The DADA2 method [43] was used to trim and denoise the data. The ASVs-based (amplicon sequence variants) clustering method was used to group the sequences, which were then classified using the SILVA classifier (v. 138) [44]. The plugins and parameters used in the processing of sequencing are provided in the supplementary materials (Table S2). The amplicon sequences identified as cyanobacterial according to the SILVA database have been verified using the Cydrasil v.2 reference alignment and the Cydrasil v.2 reference tree [45]. The alignment and mapping have been performed as described in the protocol given by Rosh and coauthors [45]. The phylogenetic placement was visualized using iTOL [46]. The raw reads are available in the BioProject database with ID SUB9183422 and the 16S rRNA gene and the 16S-23S ITS region sequences of the strains are available under the accession ID SUB9363093.

The complementary sequences of 16S and 16S-23S ITS were joined into contigs using SeqMan software (Lasergene v. 14.00, DNASTAR). The initial identification of the sequences was performed using the BLAST algorithm [47]. The multiple sequence alignment was performed using SSU-ALIGN [48]. The phylogenetic analysis was conducted based on sequences of 16S genes, which had a length of 1120 bp. The phylogenetic analysis was performed using the maximum likelihood method and 1000 replicates bootstrap in the RAxML [49]. The tree provided in this study was constructed on the basis of the GTR GAMMA model. The low numbers of reference sequences for the cyanobacteria isolated from endolithic microhabitat (crypto- and chasmoendoliths) were the reason why the 16S-23S ITS fragment was cut during the analysis and has not been analyzed further. Statistical analyses were performed using RStudio [50]. The hierarchical clustering analysis and non-metric multidimensional scaling (NMDS) have been carried out using the R environment, and the alpha diversity metrics were calculated in RStudio using “vegan”, “corrplot”, “phyloseq”, “tidyverse”, “qiime2R”, “DESeq2”, “devtools” and “FactoMineR” [50].

## 3. Results

### 3.1. Physicochemical Characteristics of Studied Mineral Substrates

The analysis of the physicochemical parameters of the soil revealed the remarkable fluctuation of studied variables within each subregion. The most stable pH values were found in the Rangkul area where the mean pH value was 7.94. In Karakul, it varied between 7.26 and 8.2 and in the Bulunkul area between 7.7 and 8.7. The electric conductivity varied much more than pH between the subregions; the lowest EC was detected for the sample F23 (Karakul) (5 µS/cm), while the highest was identified for F13 (Rangkul) (1530 µS/cm). The mean values of total nitrogen concentration calculated for subregions were lowest in Karakul (0.7 g/kg), and highest in Rangkul and Bulunkul (0.1 g/kg), while the mean values of total carbon were rising from the Bulunkul (17 g/kg) through Rangkul (19.1 g/kg) and was highest in Karakul (26 g/kg). Details concerning the values obtained for other chemical parameters of the soil such as the concentration of Fe, Na and Ca are provided in the Table S1B. 

The measured porosity varied significantly between studied samples of rocks. The mean grain size fluctuated between 3.363 (18.4 µm^2^ mean grain area; quartzite) and 14.72 µm^2^ (426.23 µm^2^ mean grain area; granite). However, the grain sizes also varied between the samples of granites and quartzites, with mean pore sizes of 7.82 µm^2^ and 6.12 µm^2^ respectively, while the mean grain size of conglomerates was a little bit bigger (8.0 µm^2^). The porosity of other rock types of the reference samples was characterized as similar to the main sample set and can be described as follows: the mean pore size of the conglomerates was similar to calcites, MGS of pegmatites in granites, the diorites, kaolinite and regolith were close to limestones. For the amphibolites, similar parameters as for the quartzes were obtained. The mean grain area (MGA) can be considered as a parameter reflecting the weathering rate of studied rocks, the highest value of which was noted for the granites (Supplementary Figure S3). The highest values of the MGA were also noted for samples collected from the highest altitudes (4111–5019). Most weathered granitic samples have been collected from the Karakul subregion.

### 3.2. The Taxonomic Composition and Structure of Endolithic and Edaphic Communities

**Figure 2:**
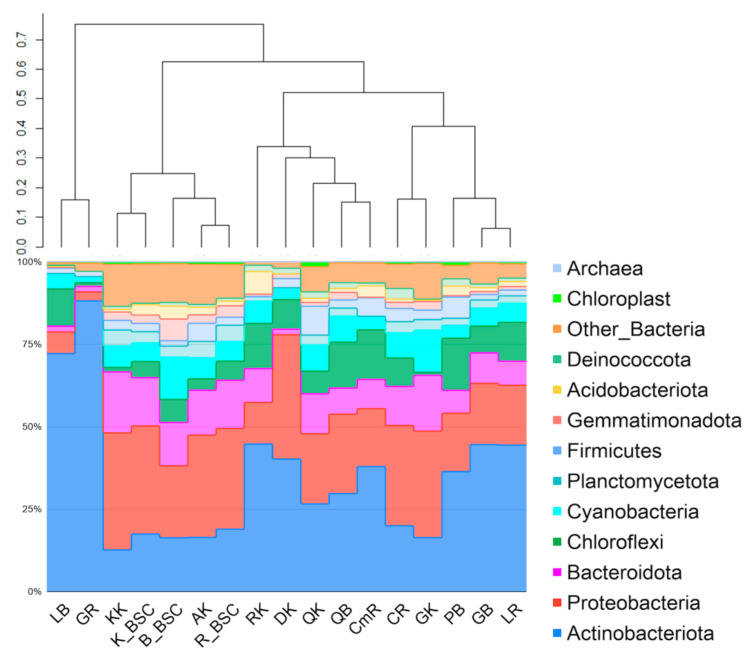
The structure of endolithic and biological soil crusts (BSCs) microbial communities based on the V3-V4 region of 16S rDNA. The sample groups’ IDs include the first letter of the rock type; the second letter was given according to the localization (GB—granite Bulunkul, PB—pegmatite Bulunkul, LB—limestone Bulunkul, QB—quartzite Bulunkul, LR—limestone Rangkul, GR—granite Rangkul, CR—calcite Rangkul, CmR—conglomerate Rangkul, GK—granite Karakul, QK—quartzite Karakul, KK—kaolinite Karakul, AK—amphibolite Karakul, DK—diorite Karakul, RK—regolith Karakul, B_BSC—from Bulunkul, R-BSC—from Rangkul, K_BSC—from Karakul). The dendrogram was made on the basis of the Bray–Curtis dissimilarity and the UPGMA (unweighted pair group method with arithmetic mean) method. The plot also includes rare reads of chloroplasts representing eukaryotic algae.

The total number of reads passed filtering obtained for endolithic metagenomes was 390,959, while for BSC the acquired amount was higher, 480,343. The number of reads and the numbers of bacterial taxa obtained for single samples of endoliths varied significantly, while the same parameters were much more stable for the BSC communities within the region. In the samples of endoliths, the number of reads per sample fluctuated between 2616 (metagenome obtained from quartzite) and 21,874 (metagenome obtained from amphibolite). The dominant phyla in endolithic microhabitats were Actinobacteria (35%, total number of reads was 144,939), Proteobacteria (22,4%, 92,768), Bacteroidetes (Bacteroidota) (10.2%, 42,612), Chloroflexi (9%, 37,580) and Cyanobacteria (5.2%). The number of reads identified as those that originated from chloroplasts accounted for an average of 0.7%. At the phylum level, the granites, quartzites, limestones, calcites and conglomerates had a similar composition (Figure 2). The clustering did not reveal a similar contribution of phyla into communities occurring in the same type of rocks from different subregions (Figure 2). The highest number of reads passed filtering was obtained for BSC sample F06 coming from the Bulunkul subregion. The lowest number of reads was also from the Bulunkul area for sample F07 (6721). The maximum cyanobacterial reads were obtained for F05 (7864) from the Bulunkul as well. The minimum was identified for sample S013 (89) from Karakul (Table S3).

The comparative analysis of the structure of both types of communities revealed the prevalence of Proteobacteria in soil-inhabiting communities (Bulunkul—21%, Rangkul—30.4% and Karakul—31.5%); with Actinobacteria being the second largest group. A higher percentage of Proteobacteria was observed only for the Karakul region.

Despite the remarkable differences in the soil chemistry identified for different sampling sites within the subregions, the relative abundance of bacterial phyla did not vary significantly. For this reason, in this study, the plot presenting the composition and structure of BSC is given for studied subregions rather than for single samples. The clustering analysis of endolithic and edaphic communities showed that communities occurring in the amphibolites and kaolinite in the Karakul subregion were more comparable to the BSCs than to other endolithic assemblages. They formed a well-defined cluster with a high level of similarity (Figure 2).

**Figure 3:**
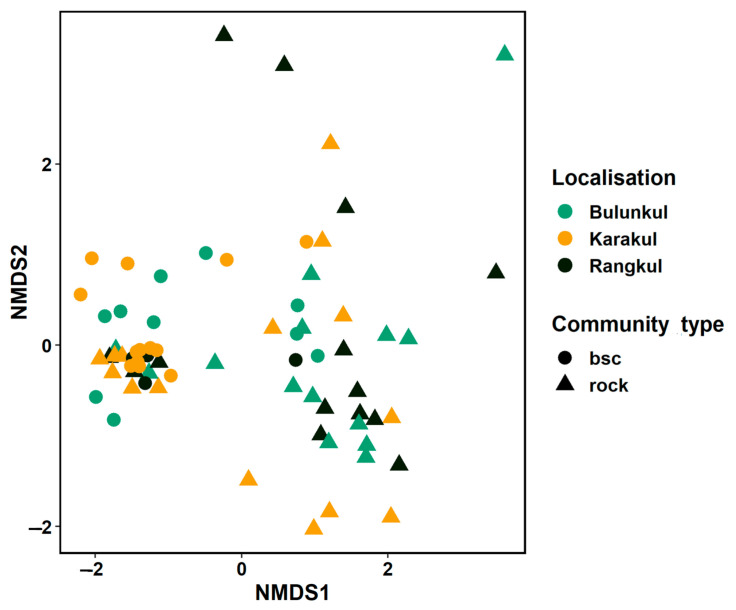
Comparison of the structure of endolithic and soil-inhabiting communities. NMDS (non-metric multidimensional scaling) using the amplicon sequence variant (ASV)-based structure of investigated communities and using the Bray–Curtis dissimilarity index.

The NMDS analysis based on the structure of both types of communities demonstrated that, in rare instances, communities grouped according to localization; this was the case for a few samples of endoliths and BSC from Bulunkul. Thus, some similarities in structure were detected for these samples. Some of the endolithic and BSC communities collected from the most arid mountainsides, and those from the area near the Uisu glacier in the Karakul subregion, and a few BSC and endoliths from the driest mountain slopes in the Rangkul area, were characterized by similar structure and were grouped in the NMDS plot (Figure 3). The endolithic communities from the same region were closely situated in the NMDS plot, and in most cases, they represented identical types of rocks.

Alpha diversity analysis performed for lithobiontic assemblages and BSC revealed that endolithic communities were characterized by slightly lower diversity. However, the variability of diversity among samples was high in endolithic communities (Figure S4). The most diverse lithobiontic bacterial communities were found in pegmatites (PK03.19B, Bulunkul), granites (G7RB, GK02.10Kh; Bulunkul) and amphibolite (AK05.48 K; Karakul). The Shannon diversity index calculated for the different communities varied between 4.45 (G3B, granite from Bulunkul) and 8.68 (PK03.19B, pegmatite from Bulunkul). Faith’s phylogenetic diversity fluctuated between 8.70 (G3B, granite from Bulunkul) and 58.42 (BK02.10 Kh, granite from Bulunkul subregion). The minimum value of Pielou’s evenness was registered for sample G29R, the granite from Rangkul (H’ = 0.81). It reached the maximum in the pegmatite sample from Bulunkul PK03.19B, calcite from Rangkul ClK04.35R, amphibolites (AK05.48K and A48bK) and calcite from Karakul (H’ = 0.96). The maximum number of observed features (observed OTU/ASV) was 513 and it was identified in the same sample (PK03.19B). The minimum number, only 44, was obtained for sample G3B (Table S3).

The BSCs’ communities demonstrated the maximum and minimum value of Pielou’s evenness index among the communities from the subregion of Karakul; with the highest value being noted in the sample F16 (J = 0.96) (close to the Lake Karakul) and the minimum value (J = 0.91) in sample F20 from the mountainside in the Karakul subregion, far away from the lake. The number of observed ASV (observed features) fluctuated between 167 (F07, Bulunkul) and 697 (F16, Karakul). Faith’s phylogenetic diversity was the lowest in F07 (PD = 25.97) and the highest in sample F16 (PD = 62.61).

The analysis of the relationships between different diversity parameters revealed a positive and statistically significant correlation between mean grain area and the number of observed features (r = 0.74, *p* < 0.05) (Figure S5A). We also observed positive, statistically significant, correlations between all diversity parameters and reads of bacteria and cyanobacteria. Spearman’s rank correlation calculated for BSCs’ communities and soil parameters revealed that among all the studied variables and diversity parameters the total carbon concentration correlated positively with bacterial reads, Faith phylogenetic diversity (r = 0.5, *p* < 0.05) and with the Shannon diversity index, and the number of observed features (observed ASVs) (r = 0.49, *p* < 0.05 for both). Other significant correlations occurred between all the diversity parameters and the number of bacterial reads. The cyanobacterial reads did not correlate significantly with these parameters or with bacterial reads. Additionally, the diversity parameters correlated positively with each other, as did some chemical elements with each other (Figure S5B). 

**Figure 4:**
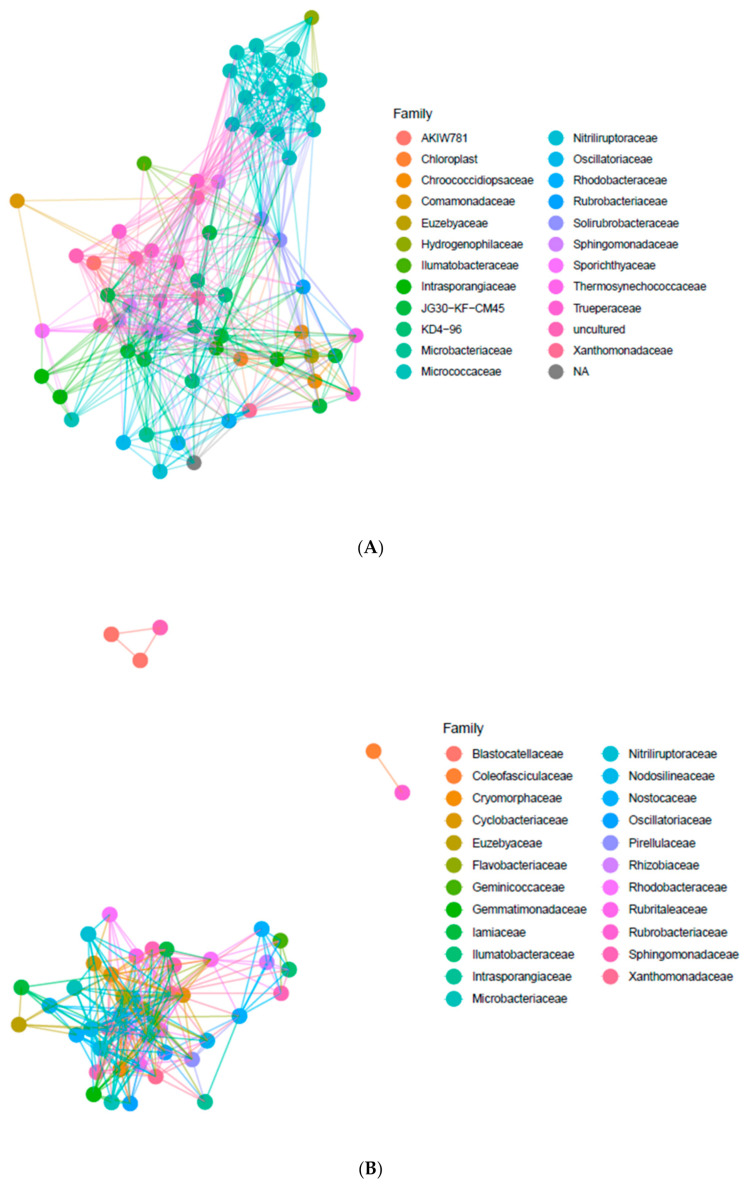
Co-occurrence of the most abundant bacterial ASVs in the endolithic microhabitat (**A**) and the BSCs (**B**). The sequence variants with a total abundance of less than 100 were removed. The distances calculated using the Bray–Curtis dissimilarity index with a maximum distance of 0.95.

The co-occurrence of bacterial taxa in endolithic and edaphic niches has been analyzed using artificial networks. Network analysis on the basis of the most abundant ASVs occurring in the endolithic niche allowed for the identification of a core community and the identification of the relationships between the most abundant families. We have noted a high contribution of Micrococcaceae, Nitriluptoraceae and Euzebyaceae in Actinobacteria, Rhodobacteriaceae, Sphingomonadaceae and Xanthomonadaceae in Proteobacteria, Truperaceae in Deinococcus-Thermus and Chroococcidiopsidaceae, Thermosynechococcaceae and Oscillatoriaceae in Cyanobacteria, and families KD 4-96, AKIW781 and JG30-KF-CM45 (Thermomicrobiales) in Chloroflexi (Figure 4A). The Micrococcaceae family was represented by the highest number of ASV, making this family the most pronounced among bacteria phyla. We also identified highly abundant families of unassigned and uncultured bacteria. The Micrococcaceae formed a separate cluster from other abundant bacterial families, while other taxa formed the larger group.

The most abundant bacterial families identified in the BSC were represented by Xanthomonadaceae and Sphingomonadaceae belonging to Proteobacteria, Flavobacteriaceae and Cytophagaceae from Bacteroidetes, Iamiaceae, Nitriliruptoraceae and Euzebyaceae belonging to Actinobacteria, Pirellulaceae belonging to Planctomycetes and finally Nodosilineaceae, Nostocaceae, Coelofasciculaceae and Oscillatoriaceae belonging to Cyanobacteria (Figure 4B). Some ASVs belonging to Coelofasciculaceae, Blastocatellaceae and Rubrobacteriaceae co-occur in the communities and were separated from other taxa. 

Comparative analysis of co-occurrence of microorganisms at the family level in endolithic and edaphic niches revealed some differences in the abundance and types of ASVs representing the same families. For example, Nodosilineaceae occurring in both types of communities was not abundant in the endolithic system. Consequently, this family is absent in the network plot performed for endolithic microbial communities.

### 3.3. The Taxonomic Composition of Cyanobacterial Communities and Relative Abundance of Identified Taxa

We observed comparatively higher heterogeneity in the composition and structure of cyanobacteria between different endolithic samples from the same area than between communities from different regions. The structure of cyanobacteria in the BSC demonstrated divergence in the communities from different regions and lower heterogeneity among samples from the same region. The highest contribution of Cyanobacteria (7.3% on average) to the endolithic community structure was found in the granites from Karakul (Figure 2). These granites were collected in sampling sites located high up in the mountains (altitudes 4111–5019 m a.s.l.). The lower contribution of cyanobacteria (5.3% on average) was observed in Bulunkul but the lowest was in Rangkul (4.9% on average) although the differences between them were small. The maximum number of cyanobacterial ASV was identified in communities inhabiting calcites from the Rangkul subregion (ClDRR, 19 ASVs), quartzites from Bulunkul QRKRKh, calcites from Rangkul Cl32RR and amphibolites from Karakul A48bK, where 17 ASVs was detected for cyanobacteria. In seven samples only one type of cyanobacterial ASVs was identified: in communities occupying granites (G3B, G4bB), pegmatites (P1B, P17Kh) from the Bulunkul subregion, calcites (ClRdR, ClD1R) from the Rangkul subregion and diorites from Karakul (D46K). Cyanobacteria contributed on average 8.1% in BSC, while chloroplasts accounted only for 0.7% on average. In contrast to endolithic communities, the contribution of Cyanobacteria in BSC was the highest in Bulunkul (12.8% on average) and lower and comparable in Rangkul (5.9% on average) and Karakul (5.6% on average). 

**Figure 5:**
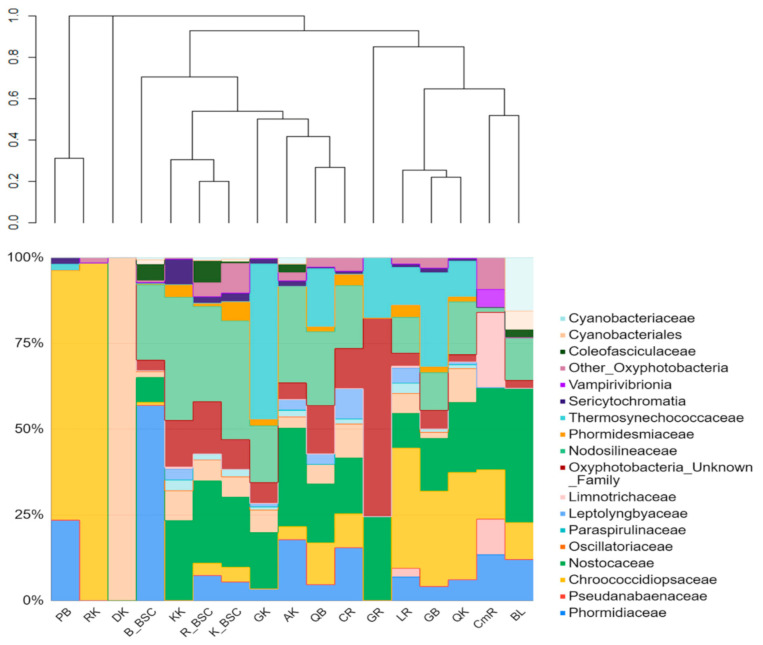
The relative abundance of cyanobacterial taxa occurring in the rocks in Eastern Pamir. The sample groups’ IDs include the first letter of the rock type. The second letter was given according to localization (GB—granite Bulunkul, PB—pegmatite Bulunkul, LB—limestone Bulunkul, QB—quartzite Bulunkul, LR—limestone Rangkul, AK—amphibolite Karakul, GR—granite Rangkul, CR—calcite Rangkul, CmR—conglomerate Rangkul, GK—granite Karakul, QK—quartzite Karakul, KK—kaolinite Karakul, DK—diorite Karakul, RK—regolith Karakul, B_BSC—biological soil crust from Bulunkul, R_BSC—from Rangkul and BSC_K—from Karakul subregion). The dendrogram was made based on the Bray–Curtis dissimilarity and UPGMA (unweighted pair group method with arithmetic mean) method.

The structure of endolithic cyanobacterial communities was characterized by high variability. The presence of the Chroococcidiopsidaceae family was a similar feature of all the rock types collected from the Bulunkul subregion. They were also identified in limestones, calcites, and conglomerates from Rangkul, and in quartzites and a coralloid from Karakul. The family Chroococcidiopsidaceae was present in 53% (23) of the studied samples of endoliths. Other abundant families were Nostocaceae, Nodosilineaceae and Thermosynechococcaceae. We identified Sericytochromatia (1.2% on average) in 8 out of 12 types of rocks. Vampirovibrionia were noted only occasionally and accounted for an exceptionally low mean percentage (0.43% on average) of the community. The cyanobacterial communities occurring in the soil in the Bulunkul subregion were characterized by the dominance of Phormidiaceae (57%) with Nodosilineaceae (22%) being the second largest group, while in the Rangkul and the Karakul communities Nodosilineaceae (27.8% and 34.6% respectively) and Nostocaceae (24% and 20.4% respectively) prevailed. Together, they accounted for more than 50% of the cyanobacteria in the communities in both subregions. However, there were also differences in the taxonomic composition and relative abundance of cyanobacterial families occurring in individual samples from studied subregions (Figure 5).

The comparative analysis of the composition of both types of communities revealed that Nodosilineaceae were detected in almost all samples of BSC and in most of the endolithic samples excluding pegmatite from Bulunkul, granites from Rangkul and diorites and regolith from Karakul. The two last samples, which were used as a reference for subsampling for this study, were represented by only one type of cyanobacterial ASVs. This was identified as *Chroococcidiopsis* in coralloid (CK) and Oscillatoriaceae (*Oscillatoria*) in diorite (DK). In terms of the SILVA classification, the sequences of *Nodosilinea* (which were ubiquitous in BSCs in Eastern Pamir) are remarkably similar to *Nodosilinea nodulosa*, which is known for its ability of nitrogen fixation. The same type of ASV was also noted in endolithic communities. Nostocaceae, which were an abundant component of both types of communities, were highly represented by the ASV identified as *Nodularia* and *Tolypothrix* (Figure 5). 

The clustering analysis of the V3-V4-based structure of both types of cyanobacterial communities revealed that endolithic communities occurring in granites and kaolinites in the Karakul region were highly similar to BSCs’ microbial consortia. Among these, the BSC from Karakul and Rangkul exhibited the highest level of similarities. Other endolithic systems were much more different from the BSC. They were also quite different from each other. The sample of kaolinite was collected from a site situated quite close to the Uisu glacier, an area that is seasonally flooded. The granites came from the same area, and from the mountain peak located on the opposite side of Lake Karakul, which is one of the largest high mountain lakes on Earth.

Isolation and cultivation of cyanobacteria from environmental samples allow for morphological and ultrastructural characterization of the microorganisms and assignment of taxonomic affiliation. The total number of isolates obtained from endolithic samples was 59. The isolates were assigned to *Chroococidiopsis* spp. (isolated from 26 samples), *Phormidium* spp. (isolated from 8 samples) and *Leptolyngbya* spp. (isolated from 8 samples). *Nodosilinea* sp. were isolated from five samples, while Nostoc-like morphotypes were isolated from four samples (Table S4). In samples from the Bulunkul subregion, *Nodosilinea* sp. prevailed in wetter locations, while the drier locations/habitats were dominated by the *Chroococcidiopsis* species. *Synechococcus* sp. was isolated from samples of granites and quartzites occurring at altitudes of 4111–4244 m a.s.l. in which the endolithic biofilms were up to 5 mm under the rock matrix, remarkably close to the upper surface of rocks.

**Figure 6:**
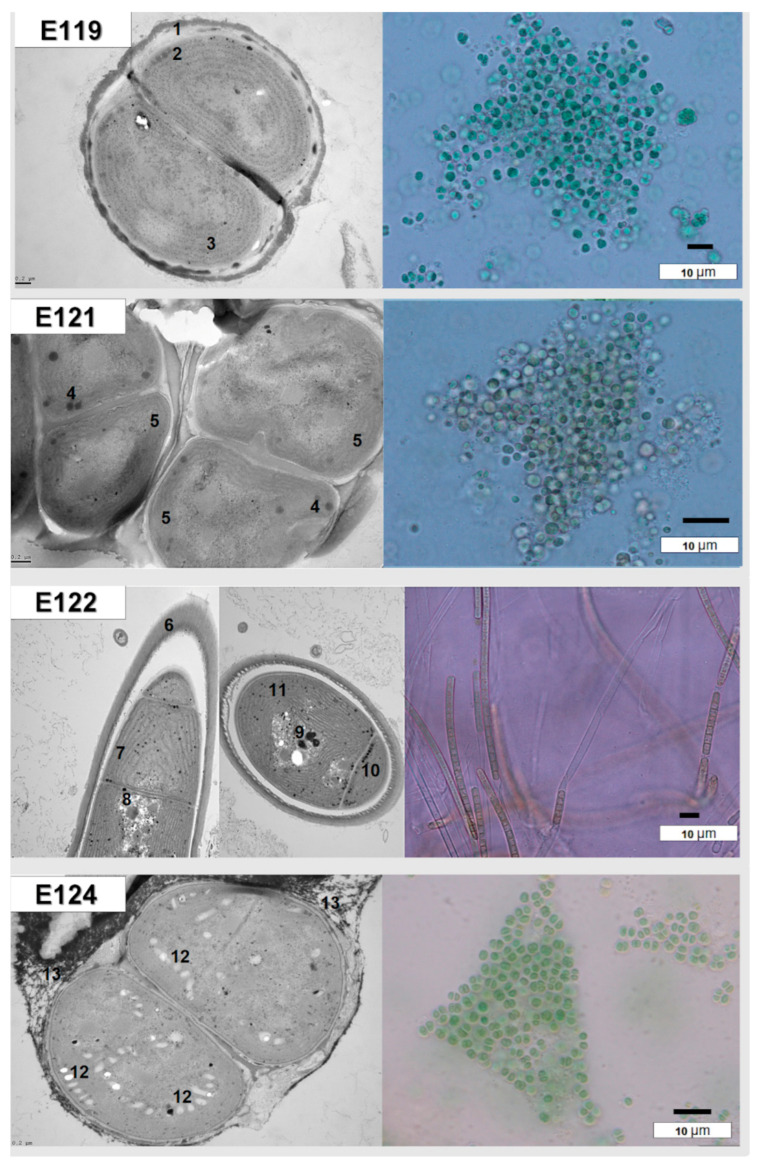
Morphology and cells’ ultrastructure of cultured cyanobacteria: (E119, E124) *Gloeocapsopsis* sp., (E121) *Chroococcidiopsis* sp. and (E122) *Wilmottia* sp. 1,13—cyanobacterial sheaths with pigment, 6—colorless cyanobacterial sheath 2,8—polyphosphates’ granules, 3,5,7—thylakoids, 4, 9—carboxysomes, 12—aerotopes, 10—granules of cyanophycin.

**Figure 7:**
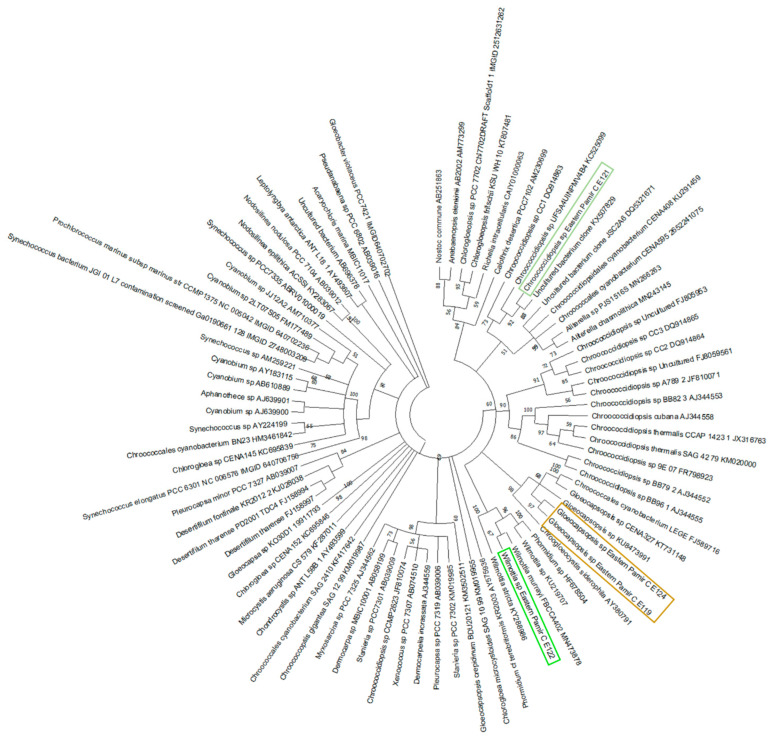
Maximum-likelihood tree based on 16S rRNA gene of cyanobacteria with the bootstrap of 1000. The sequences obtained within the present study are marked in light green (*Wilmottia* sp. Eastern Pamir CE122), brown (*Gloeocapsopsis* sp. Eastern Pamir CE119 and CE124) and dark green (*Chroococcidiopsis* sp. Eastern Pamir CE121).

The cultured endolithic strains varied in morphology and were genetically divergent. The strain CPC1 (E119) was isolated from an environmental sample of granite from Karakul (GC1K). The strain CP24R (E121) and strain CP28 (E122) were obtained from samples of limestones from the Rangkul subregion (L22R and L28R respectively), while strain CP49R (E124) came from the sample of quartzite collected from Karakul QC2K. Endolithic strains E119 (isolated from granite) and E124 (isolated from quartzite) were morphologically similar to the *Chroococcidiopsis* sp., but ultrastructural features and sequencing of partial 16S rRNA genes allowed us to assign them to the *Gloeocapsopsis* sp. (Table S5). However, these taxa were not found to be dominant in V3-V4-based 16S metabarcoding in granites (E119) and quartzites (E124) from the Karakul subregion from where the strains were isolated. In quartzites, the most frequent ASVs corresponded to Chroococcidiopsidaceae, while in granites (E119) Thermosynechococcaceae prevailed. The strain E121 of coccoid cyanobacteria and the strain E122 of filamentous non-heterocystous morphotype cyanobacteria were isolated from limestones collected from sites situated close to each other, with similar environmental conditions. The strain E121 was initially identified as *Chroococcidiopsis* sp. (Table S5; Figure 6 and Figure 7) and its morphological features resembled *Chroococcidiopsis kashayi*. The 16S metabarcoding revealed the presence of Chroococcidiopsidaceae sequences in the environmental sample (metagenome) from which the strain was isolated. However, no reference sequence of this species features in the databases. The strain E122 of the filamentous cyanobacterium was identified as *Phormidium* sp. based on the morphology, but 16S phylogeny and ultrastructure of the cells demonstrated it should be identified as *Wilmottia* sp. (Supplementary Table S5, Figure 6 and Figure 7).

## 4. Discussion

Altogether, the Pamirian endolithic communities can be considered to be more heterogeneous than those from the edaphic systems. This is in line with the currently published results of studies of microbial diversity from the cold deserts of Antarctica [12] and the Colorado Plateau [7]. It should be noted that the majority of original, published studies of endoliths focus on the communities from only a few types of rocks, sometimes from one. Our study compares colonization observed in nine different rock types, collected from different subregions. Analyzing the structure of endolithic communities at different taxonomic levels (phylum, class, family and ASV) we did not detect distribution patterns based on subregions. Nonetheless, some communities from the Karakul subregion were placed relatively close to each other. However, the NMDS ordination revealed that the structure of bacterial communities seems to be driven predominantly by the type of mineral substratum, as samples of the same type of rock from different localities appeared close. This pattern could respond to the influence of the architecture of rock (endolithic microhabitat). This is in line with other studies in which local/microenvironmental factors have been previously revealed as important drivers of endolithic colonization [6,15].

Despite the use of different markers, the similarities in community structure of endoliths at the higher taxonomic level and alpha-diversity indices were found to be similar to those previously reported in Antarctica [16,17,20], high-altitude tundra of Tibet [51] and in the Colorado Plateau [7]. Studies comparing the composition and structure of bacterial communities occurring in endolithic and edaphic systems are scarce. The tendency of higher relative abundance of Proteobacteria in the BSC and endolithic assemblages dominated by Actinobacteria, which was observed in this study, was also found in the cold desert of Antarctica [12,16]. The relative abundance of Actinobacteria in BSC communities analyzed within this study was higher in more oligotrophic soils, which were connected, for instance, with a larger distance to water reservoirs. These patterns were also found for endolithic and hypolithic assemblages occurring in the polar desert of Antarctica [16,20] and the Atacama Desert [15].

In the communities analyzed here, the Actinobacteria family was mostly represented by Euzebyaceae. Another abundant family, which was represented in both types of communities, was the Xanthomonadaceae, which belongs to Proteobacteria and is known for its N-fixing ability. Rhizobiaceae, another family with common diazotrophic abilities, was in turn abundant only in the BSCs. The high contribution of these families to the soil-inhabiting bacteria was also reported by other researchers studying samples from Antarctica [12,52].

Differences concerning the relative abundance of dominant bacterial phyla identified within the present study, and in previously reported investigations, can be the result of divergent environmental conditions (e.g., temperature, light conditions and altitude) and geographic isolation. As has been observed in other studies, cyanobacterial communities in the endolithic niche were represented by coccoid package-forming *Chroococcidiopsis* [6,20,51,53] *Gloeocapsopsis* [54], filamentous, non-heterocystous Trichocoleusaceae and Coleofasciculaceae and filamentous, heterocystous Nostocaceae [54]. The edaphic niche in turn was dominated by filamentous non-heterocystous Nodosilineaceae and Phormidiaceae [7]. The structure of lithobiontic cyanobacterial communities at the family level did not demonstrate the pattern that reflects the geographical regionality (studied subregions) or type of rocks.

The high throughput sequencing technology (next-generation sequencing) provided the opportunity to explore cyanobacterial diversity without applying a culture-dependent approach. However, the isolation and cultivation of cyanobacteria is still an important step to optimize the method of studying this group of microorganisms. In the present study four cultured endolithic cyanobacterial taxa have been characterized following the polyphasic approach for endolithic taxa of cyanobacteria. These cyanobacterial taxa are characterized by high morphological variability, especially those isolated from the most extreme environments. The presence of *Chroococcidiopsis* sp. and *Gloeocapsopsis* sp. was confirmed using phylogenetic analysis of the 16S rRNA gene sequences of these cyanobacterial cultures, similarly as for cyanobacterial communities from the Atacama Desert [54]. Another isolated strain was identified based on the morphology as *Wilmottia* sp. Its closes relative identified on the basis of the 16S rRNA is *Wilmottia murrayi*, which belongs to Oscillatoriales and which was reported from Antarctica [55]. The results of culture-dependent methods additionally used in this study illustrated that Pamirian endolithic cyanobacterial communities need to be more deeply explored in further works. These regions can be considered as potential sources of novel species or species that lack reference sequences in the databases.

Our results demonstrated that the hypothesis concerning the separation of the bacteria occurring in the edaphic and lithobiontic niches [2,7,12,20] cannot be in extenso implemented to describe the distribution of bacteria in endolithic systems and biological soil crusts in Eastern Pamir. Primarily, similar patterns identified in the distribution of bacterial taxa at the endolithic and edaphic niches in Eastern Pamir were not observed for all of the studied samples and the three studied subregions. Structure-based NMDS analysis clearly indicated that some of the biocrusts and endolithic communities were placed together due to the high degree of similarity of the abundance of bacterial taxa. The strong relation between the composition and structure of microbial communities from both niches was observed for samples from the Karakul and Rangkul subregions, which were collected from very dry and oligotrophic places. The rocks in these areas were attached to the soil, or even partially covered by soil, and were strongly weathered, which can favor soil particles occupying rock pores, thus facilitating the connectivity. For example: on the Uisu glacier (Karakul subregion), biocrusts from this oligotrophic area [30] and kaolinite and amphibolites samples of relatively small size and attached to the soil, were collected from nearby the glacier. The melting of the glacier in this area can create an occasional connection via water between the endolithic communities attached to the soil and the BSC. Such similarities between soil communities and the endolithic assemblages were also reported for the communities from Victoria Valley of Antarctica, where the sampling was performed during the austral summer season of January when the soil moisture increases due to the snow melting [20]. The connection between the two types of Pamirian microbial communities was also illustrated by the presence of identical types of ASV, represented by Nodosilineacea and certain Nostocaceae taxa, in the majority of soil and many rock communities. These results suggest that while similarities between structures of edaphic and endolithic communities are mainly determined by the proximity and physical connection between rocks and BSC, as in the case of Karakul samples, the presence of taxa characteristic of the BSC in the highly specialized communities of endoliths seems to occur more often. Despite this fact, however, the endolithic communities remain very distinct, having divergent structure, as they are shaped by other microenvironmental factors.

Diversity metrics is an informative characteristic of bacterial communities, which characterizes environmental conditions such as the trophic level or water availability [54]. The correlation of mean grain area (microstructural factor) with the number of observed ASVs in the endolithic communities, which was noted in our study, can support the hypothesis concerning the importance of rock architecture for microbial diversity inhabiting the rocks [6,15,53]. The remarkable differences in the soil chemistry noted for samples from the same subregion suggested that there could be dissimilarities in the taxonomic structure of BSC; however, the structure of the communities was similar within the region. Among all the studied factors, the total carbon concentration level in the soil seemed to have the greatest influence on the diversity of bacteria in BSC. This was illustrated by a statistically significant, positive correlation obtained for the observed features, bacterial reads and Faith’s PD. In this way, soils with higher carbon content may support more diverse and richer communities. The high percentage of cyanobacteria from families such as Chroococcidiopsidaceae, Nostocaceae and Nodosilineaceae, which are known for their abilities to fix atmospheric nitrogen, suggest their particular importance in the environment characterized by low content of nitrogen [27,56,57,58]. However, the correlation between specific family or taxon, and the nitrogen concentration has not been found. The role of *Chroococcidiopsis* in loess formation due to its ability to produce the high biomass of extracellular polymeric substances, which was reported recently [58], is another reason to explore this genus and its involvement in the biogeochemical process occurring in the desert ecosystem. The results of the present study suggest that an interesting aspect, which can be explored in further works, is the seasonal fluctuation (with a few years of coverage) in the relative abundance of cyanobacteria and the C and N content in the soil. However, due to logistic difficulties such seasonal sampling was not possible in the present study. For this reason, this investigation was performed during the vegetation season, allowing coverage of the diversity of bacteria occurring at both lithobiontic and edaphic niches.

## 5. Conclusions

The composition and structure of endolithic communities occurring in the mountain desert of Pamir is mainly shaped by specific microenvironmental factors, which was illustrated by the heterogeneous distribution of taxa within each niche. A link between endolithic and edaphic communities was established, particularly in the most oligotrophic areas, which are characterized by the most severe climatic conditions. While the structure of soil-inhabiting bacterial communities was characteristic for the different subregions of the Eastern Pamir, no patterns associated with geographical distribution were detected for bacterial endolithic communities. The composition and structure of endolithic communities in these geologically divergent areas cannot be associated purely with their geographical location. It seems to be connected more strongly to the specific physicochemical features of different rock types. The cyanobacterial communities occurring at edaphic and endolithic niches in Eastern Pamir share similarities with these from other arid and hyperarid environments; however, the assemblages are also characterized by particular features associated with the unique climatic conditions found in the studied subregions of Pamir.

## Data Availability

The raw reads are available in the BioProject database with ID SUB9183422 and the 16S rRNA gene and the 16S-23S ITS region sequences of the strains are available under the accession ID SUB9363093. The phylogenetic placement of the V3-V4 sequences is available online under the link https://itol.embl.de/tree/2128710254124841612883374 (accessed on 8 April 2021), Login: nkhomutovska, password: rGDlKYQa.

## Supplementary Materials

The following are available online at https://www.mdpi.com/article/10.3390/biology10040314/s1. Figure S1. Studied area. The study areas that are marked in blue belong to Bulunkul, Rangkul and Karakul subregion. Figure S2. The rarefaction curve showing the sequencing. Figure S3. The box plot shows the fluctuation of the mean grain size (MGS) and mean grain area (MGA) for the samples of endoliths, excluding the reference samples due to their limited numbers. Figure S4. The alpha diversity metrics calculated for communities inhabiting different types of rock reflected various microhabitats. Figure S5. The correlation plots reflect the relationships between the alpha diversity metrics and selected environmental parameters. Table S1. The geographic location, rock characterization (**A**) BSC geographic location as well as and chemistry of the soil (**B**). Table S2. Scripts used for analyses of sequences in QIIME2 (version 2020.11). Table S3. The alpha diversity metrics calculated for studied communities. Table S4. Comparative analysis of endolithic cyanobacterial communities based on culture-dependent and culture-independent approach. Table S5. The description of analyzed cyanobacterial strains.
