# Supplementary figures and images for: Connectivity of Edaphic and Endolithic Microbial Niches in Cold Mountain Desert of Eastern Pamir (Tajikistan)
Source: Biology (Basel). 2021 Apr 9;10(4):314. doi: 10.3390/biology10040314 (PMC8069199; doi:10.3390/biology10040314)

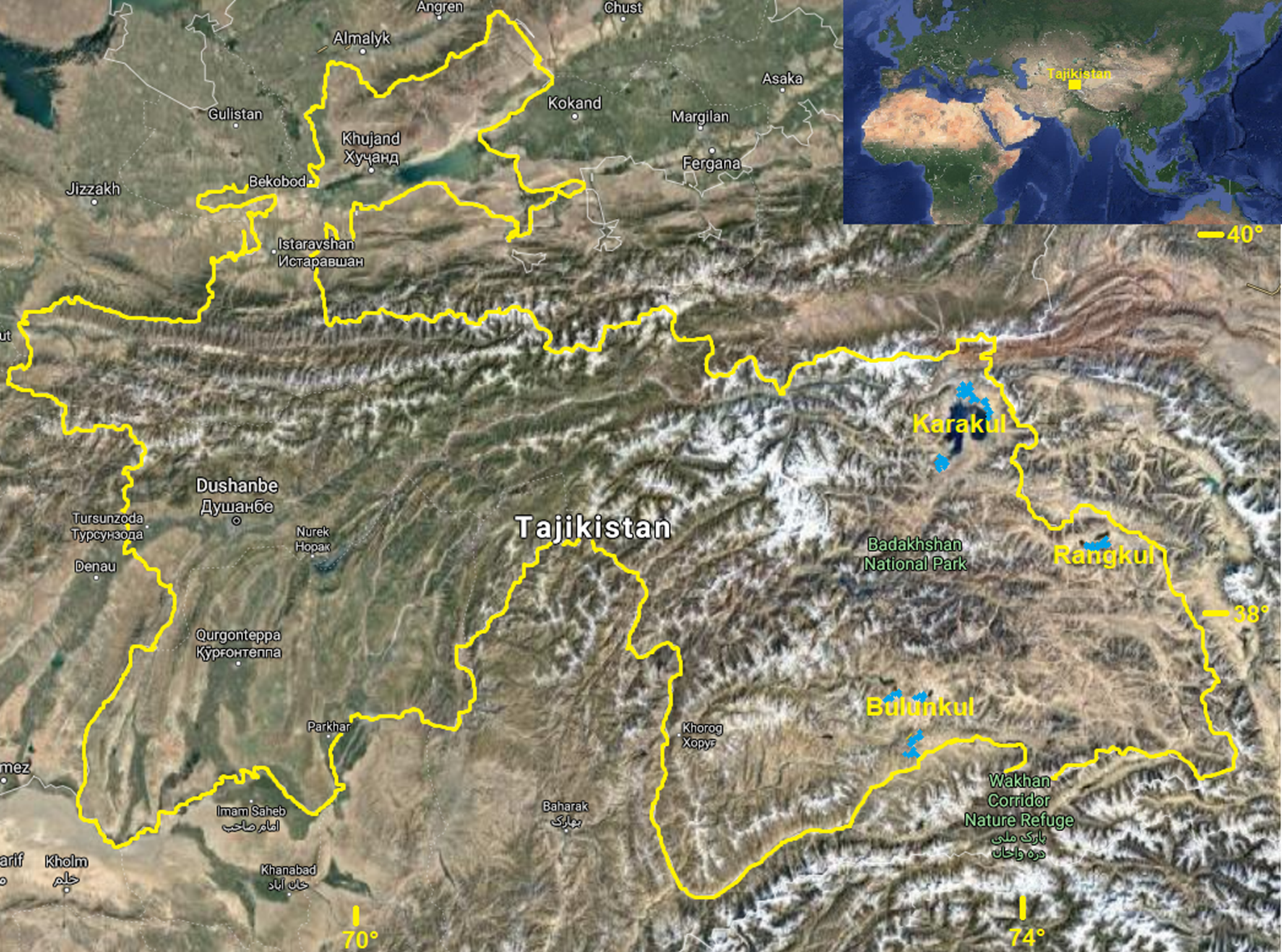

Supplement: Supplementary file 1 [file biology-10-00314-s001.zip › Fig. S1.TIF]

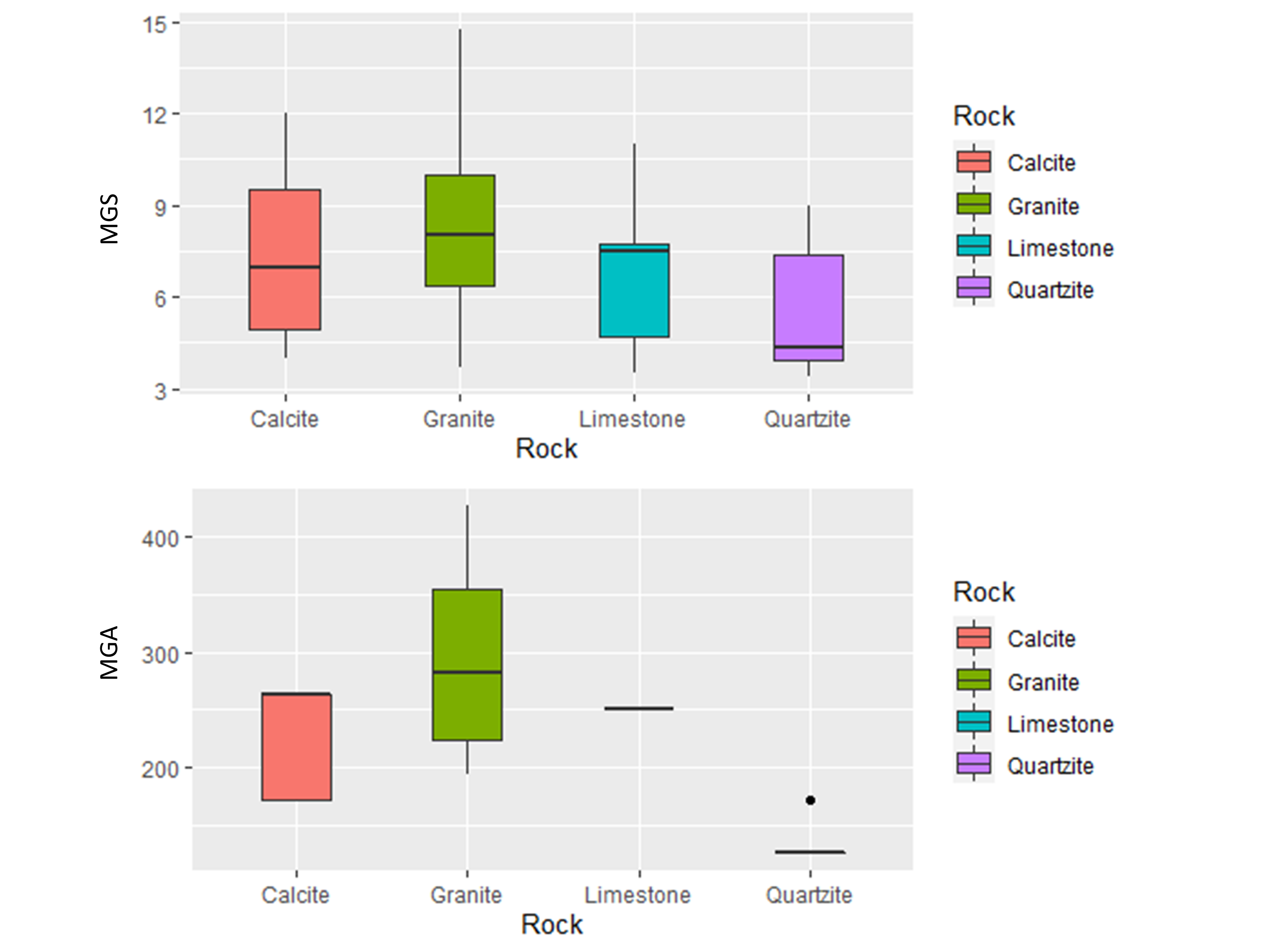

Supplement: Supplementary file 1 [file biology-10-00314-s001.zip › Fig. S3.TIF]

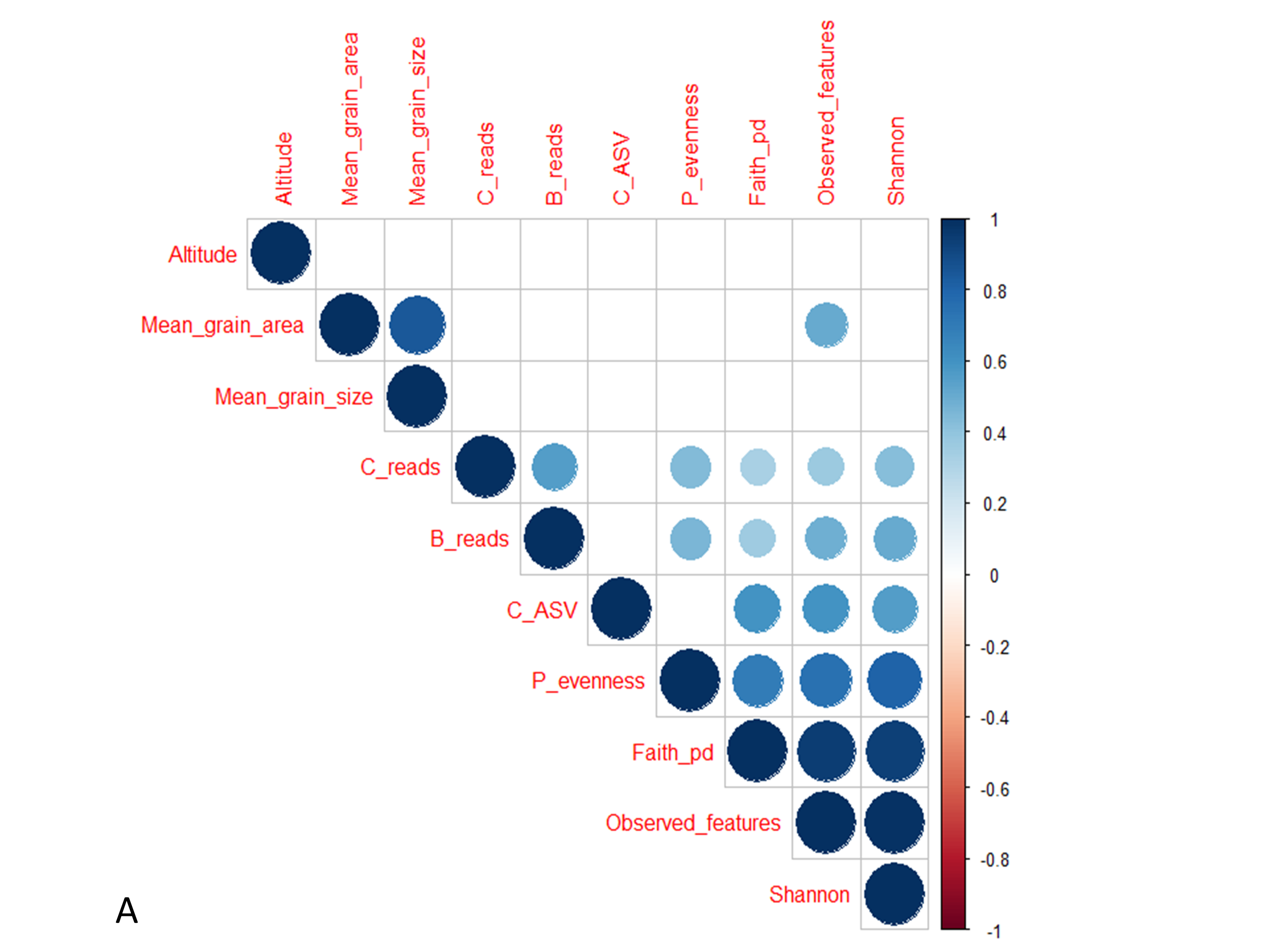

Supplement: Supplementary file 1 [file biology-10-00314-s001.zip › Fig. S5A.tif]

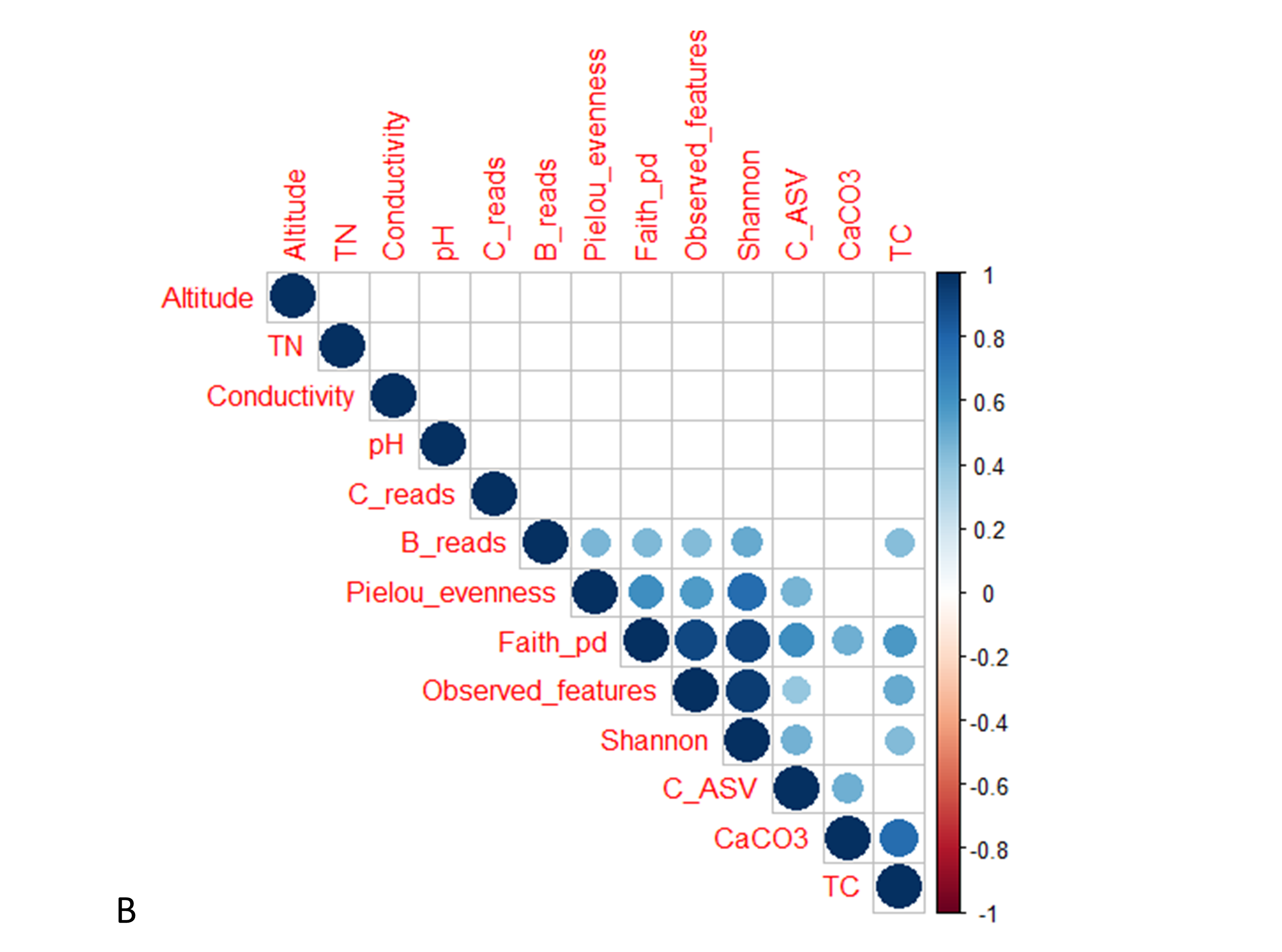

Supplement: Supplementary file 1 [file biology-10-00314-s001.zip › Fig. S5B.tif]

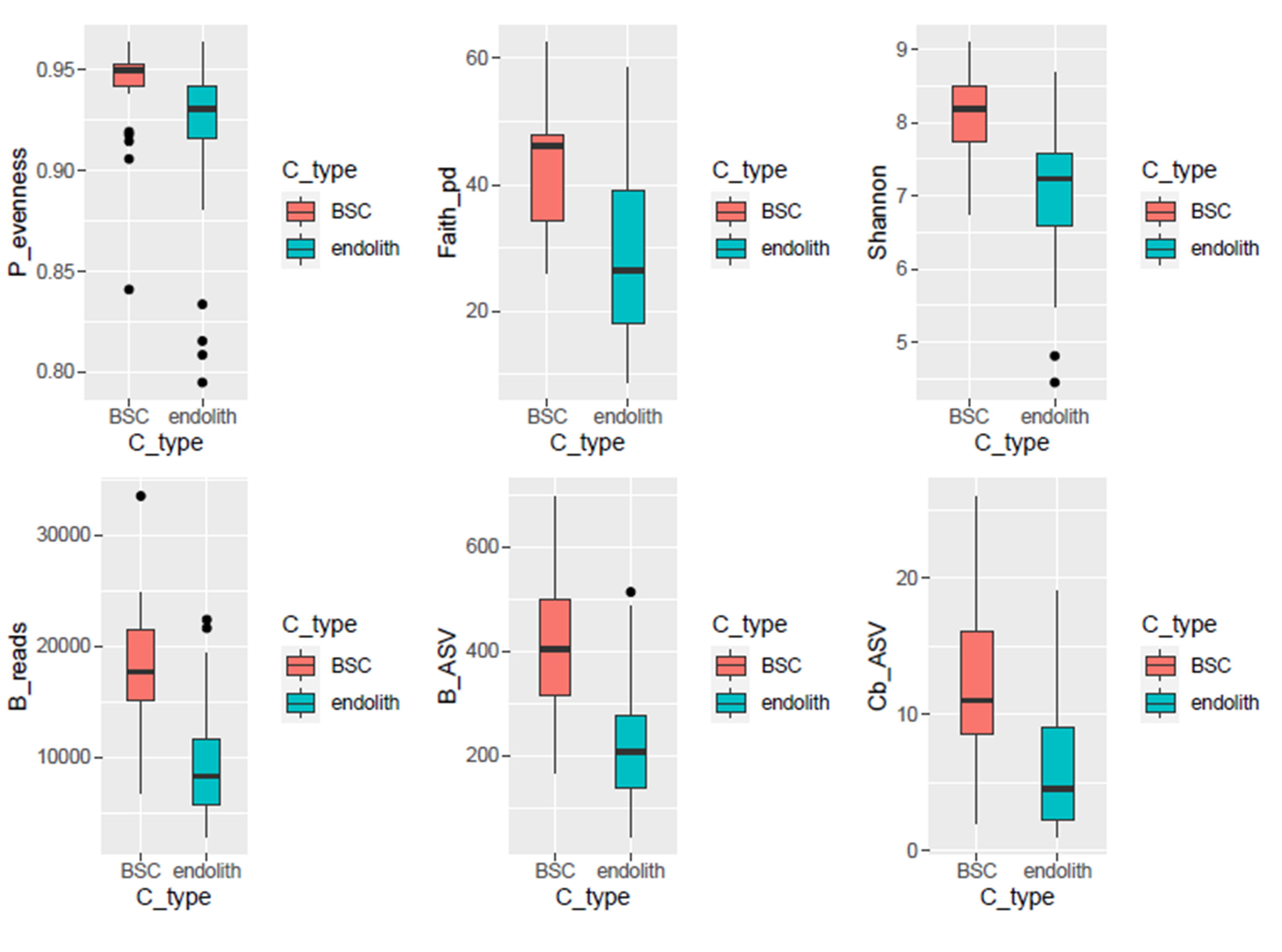

Supplement: Supplementary file 1 [file biology-10-00314-s001.zip › Fig. S4. The alpha diversity metrics.tif]

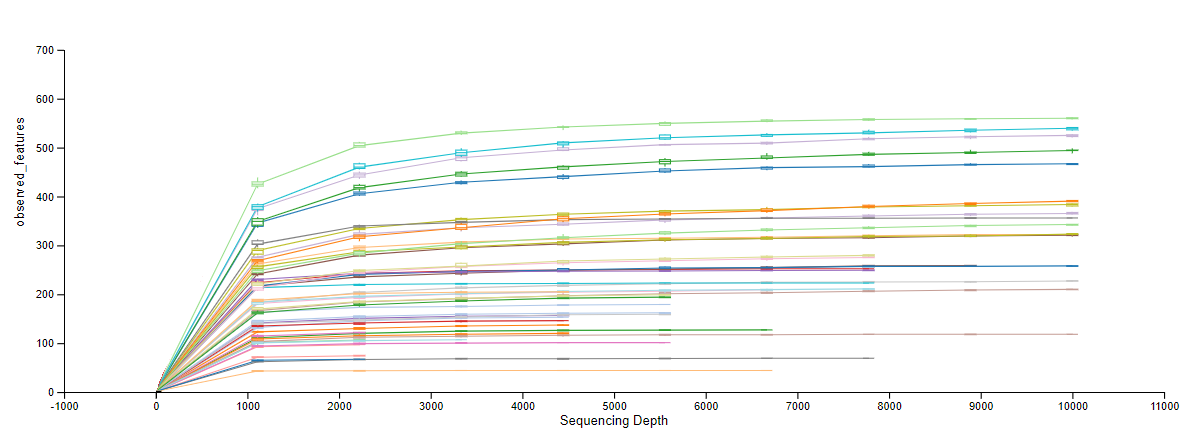

Supplement: Supplementary file 1 [file biology-10-00314-s001.zip › Fig S2. The rarefaction.tif]
