# Supplementary material for: Connectivity of Edaphic and Endolithic Microbial Niches in Cold Mountain Desert of Eastern Pamir (Tajikistan)
Source: Biology (Basel). 2021 Apr 9;10(4):314. doi: 10.3390/biology10040314 (PMC8069199; doi:10.3390/biology10040314)
Supplement: Supplementary file 1 [file biology-10-00314-s001.zip › Supplementary Tables Biology-1160517.doc]

**Table S1**. The geographic location, rock characterization (A) and approximate geographic location of the BSCs as well as chemistry of the soil (B).

(A)

| **Sample ID** | **Localization** | **Rock type** | **Latitude** | **Longitude** | **Altitude^1^** | | |
| --- | --- | --- | --- | --- | --- | --- | --- |
| **G2B** | Bulunkul | granite | N37⁰29.279' | 072⁰48.015 | | 4226 |  |
| **G3B** | Bulunkul | granite | N37⁰29.279' | 072⁰48.016 | | 4226 |  |
| **G4bB** | Bulunkul | granite | N37⁰29.279' | 072⁰48.018 | | 4226 |  |
| **G7RB** | Bulunkul | granite | N37⁰42.365' | 072⁰58.397' | | 3712 |  |
| **G12bKh** | Bulunkul | granite | N37⁰26.468' | 073⁰04.062' | | 4314 |  |
| **GK02.10Kh** | Bulunkul | granite | N37⁰26.455' | 073⁰04.521' | | 4244 |  |
| **G20Kh** | Bulunkul | granite | N37⁰33.136' | 073⁰06.608' | | 3917 |  |
| **L4B** | Bulunkul | limestone | N37⁰29.279' | 072⁰48.017 | | 4226 |  |
| **L14aKh** | Bulunkul | limestone | N37⁰26.478' | 073⁰04.020' | | 4337 |  |
| **P1B** | Bulunkul | pegmatite | N37⁰29.279' | 072⁰48.014 | | 4226 |  |
| **PK03.19Kh** | Bulunkul | pegmatite | N37⁰26.363' | 073⁰04.700' | | 4244 |  |
| **P17Kh** | Bulunkul | pegmatite | N37⁰26.363' | 073⁰04.700' | | 4244 |  |
| **QKKRKh** | Bulunkul | quartzite | N37⁰26.363' | 073⁰04.700' | | 4244 |  |
| **QRKRHh** | Bulunkul | quartzite | N37⁰26.363' | 073⁰04.700' | | 4244 |  |
| **L21R** | Rangkul | limestone | N38⁰26.009' | 074⁰17.352' | | 3921 |  |
| **L22R** | Rangkul | limestone | N38⁰25.965' | 074⁰17.314' | | 3932 |  |
| **L23R** | Rangkul | limestone | N38⁰25.959' | 074⁰17.305' | | 3950 |  |
| **L26R** | Rangkul | limestone | N38⁰26.009' | 074⁰17.429' | | 3901 |  |
| **L28R** | Rangkul | limestone | N38⁰26.009' | 074⁰17.429' | | 3901 |  |
| **L34R** | Rangkul | limestone | N38⁰24.892' | 074⁰11.325' | | 3919 |  |
| **G29R** | Rangkul | granite | N38⁰26.009' | 074⁰17.429' | | 3901 |  |
| **ClK04.35R** | Rangkul | calcite | N38⁰24.892' | 074⁰11.325' | | 3919 |  |
| **Cl1RdR** | Rangkul | calcite | N38⁰24.892' | 074⁰11.325' | | 3919 |  |
| **ClD1R** | Rangkul | calcite | N38⁰24.892' | 074⁰11.325' | | 3919 |  |
| **ClDRR** | Rangkul | calcite | N38⁰24.892' | 074⁰11.325' | | 3919 |  |
| **Cl32RR** | Rangkul | calcite | N38⁰26.386' | 074⁰08.644' | | 3765 |  |
| **C37R** | Rangkul | conglomerate | N38⁰24.506' | 074⁰11.330' | | 4109 |  |
| **C38R** | Rangkul | conglomerate | N38⁰24.450' | 074⁰11.392' | | 4164 |  |
| **C39R** | Rangkul | conglomerate | N38⁰24.459' | 074⁰11.252' | | 4129 |  |
| **Q50K** | Karakul | quartzite | N39⁰17.035' | 073⁰18.483' | | 4025 |  |
| **QK06.50bK** | Karakul | quartzite | N39⁰17.035' | 073⁰18.483' | | 4025 |  |
| **Q45K** | Karakul | quartzite | N39⁰17.562' | 073⁰16.718' | | 4105 |  |
| **QE014K** | Karakul | quartzite | N39⁰19.502' | 073⁰11.771' | | 4362 |  |
| **Q49bK** | Karakul | quartzite | N39⁰17.046' | 073⁰18.519' | | 4021 |  |
| **QC2K** | Karakul | quartzite | N39⁰17.560' | 073⁰16.685' | | 4111 |  |
| **A48bK** | Karakul | amphibolite | N39⁰17.040' | 073⁰18.544' | | 4022 |  |
| **AK05.48K** | Karakul | amphibolite | N39⁰17.040' | 073⁰18.544' | | 4022 |  |
| **GC1K** | Karakul | granite | N39⁰17.551' | 073⁰16.716' | | 4114 |  |
| **G53K** | Karakul | granite | N39⁰17.560' | 073⁰16.685' | | 4111 |  |
| **G54RK** | Karakul | granite | N38⁰50.660' | 073⁰23.644' | | 5019 |  |
| **D46K** | Karakul | diorite | N39⁰17.551' | 073⁰16.716' | | 4114 |  |
| **R52K** | Karakul | regolith | N39⁰08.017' | 073⁰26.437' | | 3916 |  |
| **K44K** | Karakul | kaolinite | N39⁰19.502' | 073⁰11.771' | | 4362 |  |

**(B)**

| **Sample ID** | **Localization** | **Altitude^1^** | **Latitude** | **Longitude** | **Fe^2^** | **Mg_tot^3^** | **Na_tot^3^** | **Ca_tot^3^** | **EC^4^** | **pH** | **TN^5^** | **TC^5^** |
| --- | --- | --- | --- | --- | --- | --- | --- | --- | --- | --- | --- | --- |
| **B01** | Bulunku | NA | NA | NA | NA | NA | NA | NA | NA | NA | NA | NA |
| **F01** | Bulunkul | 3712 | N37⁰42.365' | 072⁰58.397' | 1.36 | 0.78 | 0.60 | 0.78 | 6.55 | 8.1 | 2.3 | 34.1 |
| **F02** | Bulunkul | 3712 | N37⁰42.365' | 072⁰58.397' | 1.62 | 0.18 | 0.01 | 0.04 | 0.32 | 7.9 | 0.3 | 3.9 |
| **F03** | Bulunku | 3793 | N37⁰40.669' | 073⁰10.670' | 0.51 | 0.32 | 0.04 | 0.80 | 1.22 | 7.9 | 0.4 | 16.1 |
| **F04** | Bulunku | 3793 | N37⁰40.669' | 073⁰10.670' | 0.57 | 0.31 | 0.44 | 0.22 | 11.41 | 8.3 | 0.4 | 11.1 |
| **F05** | Bulunku | 3793 | N37⁰40.669' | 073⁰10.670' | 0.71 | 0.11 | 0.00 | 0.08 | 0.39 | 8.2 | 0.5 | 8.2 |
| **F06** | Bulunku | 3793 | N37⁰40.669' | 073⁰10.670' | 1.28 | 0.51 | 0.14 | 0.58 | 3.76 | 7.9 | 2.8 | 42.5 |
| **F07** | Bulunku | 4244 | N37⁰26.363' | 073⁰04.700' | 1.07 | 0.67 | 0.47 | 0.10 | 0.40 | 7.9 | 0.7 | 11.1 |
| **F08** | Bulunku | 4260 | N37⁰26.455' | 073⁰04.521' | 1.60 | 0.24 | 0.06 | 0.29 | 1.69 | 7.7 | 1.8 | 22.9 |
| **F09** | Bulunku | 3917 | N37⁰33.136' | 073⁰06.608' | 0.38 | 0.15 | 0.11 | 0.13 | 4.13 | 7.6 | 0.1 | 3.8 |
| **F10** | Rangkul | 3921 | N38⁰26.009' | 074⁰17.352' | 1.54 | 0.41 | 0.03 | 0.77 | 2.18 | 7.7 | 0.4 | 16.8 |
| **F11** | Rangkul | 3921 | N38⁰26.009' | 074⁰17.352' | 2.78 | 0.87 | 0.24 | 1.10 | 11.08 | 7.9 | 2.0 | 30.0 |
| **F12** | Rangkul | 3921 | N38⁰26.009' | 074⁰17.352' | 1.42 | 0.15 | 0.34 | 0.04 | 0.31 | 7.9 | 01.0 | 11.9 |
| **F13** | Rangkul | 4015 | N38⁰26.386' | 074⁰08.644' | 1.85 | 0.71 | 0.10 | 1.52 | 15.30 | 7.9 | 0.6 | 17.7 |
| **F14** | Karakul | 4025 | N39⁰17.046' | 073⁰18.519' | 1.39 | 0.71 | 0.10 | 2.03 | 10.79 | 8.1 | 0.8 | 39.3 |
| **F15** | Karakul | 4025 | N39⁰17.046' | 073⁰18.519' | 1.49 | 0.81 | 0.06 | 1.67 | 2.59 | 8.1 | 0.8 | 44.6 |
| **F16** | Karakul | 4025 | N39⁰17.046' | 073⁰18.519' | 1.21 | 0.50 | 0.09 | 1.36 | 0.97 | 8.1 | 1.1 | 51.7 |
| **F17** | Karakul | 4111 | N39⁰17.560' | 073⁰16.685' | 1.39 | 0.17 | 0.10 | 2.03 | 9.17 | 8.7 | 0.1 | 17.2 |
| **F18** | Karakul | 4362 | N39⁰19.502' | 073⁰11.771' | 1.494 | 0.81 | 0.06 | 1.67 | 2.59 | 8.1 | 0.1 | 5.2 |
| **F19** | Karakul | 4362 | N39⁰19.502' | 073⁰11.771' | 1.21 | 0.50 | 0.09 | 1.36 | 3.91 | 7.7 | 0.3 | 25.4 |
| **F20** | Karakul | 4362 | N39⁰19.502' | 073⁰11.771' | 1.35 | 0.52 | 0.10 | 1.19 | 0.70 | 7.9 | 0.4 | 35.6 |
| **F21** | Karakul | 4362 | N39⁰19.502' | 073⁰11.771' | 1.43 | 0.50 | 0.09 | 0.81 | 1.14 | 7.9 | 0.3 | 31.3 |
| **F22** | Karakul | 3916 | N39⁰08.017' | 073⁰26.437' | 0.08 | 0.52 | 0.09 | 0.60 | 4.79 | 8.1 | 0.5 | 13.5 |
| **F23** | Karakul | 3916 | N39⁰08.017' | 073⁰26.437' | 1.86 | 0.59 | 0.92 | 0.68 | 0.05 | 7.8 | 3.3 | 9.0 |
| **F24** | Karakul | 3916 | N39⁰08.017' | 073⁰26.437' | 2.39 | 0.47 | 0.07 | 1.09 | 5.65 | 8.1 | 0.5 | 15.5 |
| **F25** | Karakul | NA | NA | NA | NA | NA | NA | NA | NA | NA | NA | NA |
| **S013** | Karakul | NA | NA | NA | NA | NA | NA | NA | NA | NA | NA | NA |

^1^ Altitude - m a.s.l., ^2^Fe – iron concentration in g/kg, ^3^Mg_tot, Na_tot, Ca_tot – the concentrations of total Mg, Na and Ca in the soil given in mol/kg, ^4^EC - electric conductivity of the soil in μS/cm-, ^5^TN, ^5^TC – total nitrogen (TN) and total carbon (TC) concentration in g/kg.

**Table S2**. Scripts used for analyses of sequences in QIIME2 (version 2020.11)

\# import data

qiime tools import

--type 'SampleData[PairedEndSequencesWithQuality]'

--input-path Rocks/

--input-format CasavaOneEightSingleLanePerSampleDirFmt

--output-path demux-paired-end.qza

\# visualisation

qiime demux summarize

--i-data demux-paired-end.qza

--o-visualization demux-paired-end.qzv

\# Denoise and dereplicate paired-end sequences. This method denoises paired-end sequences, dereplicates them, and filters chimeras

qiime dada2 denoise-paired

--i-demultiplexed-seqs demux-paired-end.qza

--p-trim-left-f 5

--p-trim-left-r 5

--p-trunc-len-f 300

--p-trunc-len-r 300

--o-table table.qza

--o-representative-sequences rep-seqs.qza

--o-denoising-stats denoising-stats.qza

\# Assign taxonomy using Silva 138 99% OTUs (full-length, seven-level taxonomy) classifier

qiime feature-classifier classify-sklearn

--i-classifier silva-138-99-nb-classifier.qza

--i-reads rep-seqs.qza

--o-classification taxonomy.qza

#Filtering of Cyanobacterial sequences

qiime taxa filter-seqs

--i-sequences rep-seqs.qza

--i- taxonomy taxonomy.qza

--p-include Cyanobacteria

--o-filtered-sequences rep-seqs_Cyanobacteria

#Filter Cyanobacterial sequences

qiime taxa filter-seqs

--i-sequences rep-seqs.qza

--i-taxonomy taxonomy-without-spaces.qza

--p-include Cyanobacteria

--o-filtered-sequences rep-seqs_Cyanobacteria_Seqs-all-2019_

\# Assign taxonomy using Silva 138 99% OTUs

qiime feature-classifier classify-sklearn

--i-classifier silva-138-99-nb-classifier.qza

--i-reads rep-seqs_Cyanobacteria.qza

--o-classification taxonomy_Cyanobacteria.qza

\# Merge taxonomy and table with numbers of futures

qiime taxa collapse

--i-table table.qza

--i-taxonomy taxonomy_Cyanobacteria.qza

--p-level 7

--o-collapsed-table collapsed_Cyanobacteria

#Convert biom file

biom convert

--input-fp feature-table biom

--o Collapsed_taxonomy.tsv

--to-tsv

#Visualize result

qiime taxa barplot

--i-table table.qza

--i-taxonomy taxonomy.qza

--o-visualization Viz_Seqs

**Table S3**. The alpha diversity metrics calculated for studied communities.

| **Sample ID** | **C_type^1^** | **P_evenness^2^** | **Faith_pd^3^** | **B_ASV^4^** | **Shannon^5^** | **Cb_ASV^6^** | **Cb reads^7^** | **B_reads^8^** |
| --- | --- | --- | --- | --- | --- | --- | --- | --- |
| **G2B** | endolith | 0.91 | 29.30 | 201 | 7.00 | 2 | 73 | 10744 |
| **G3B** | endolith | 0.82 | 8.70 | 44 | 4.45 | 1 | 69 | 7723 |
| **G4bB** | endolith | 0.88 | 13.85 | 108 | 5.95 | 1 | 11 | 5735 |
| **G7RB** | endolith | 0.95 | 47.80 | 485 | 8.48 | 16 | 1269 | 21608 |
| **G12bKh** | endolith | 0.93 | 19.58 | 176 | 6.97 | 2 | 93 | 6480 |
| **GK02.10Kh** | endolith | 0.94 | 58.42 | 405 | 8.11 | 10 | 1354 | 12053 |
| **G20Kh** | endolith | 0.93 | 17.02 | 149 | 6.68 | 6 | 609 | 5419 |
| **L4B** | endolith | 0.79 | 16.70 | 119 | 5.48 | 9 | 562 | 11944 |
| **L14aKh** | endolith | 0.94 | 16.02 | 129 | 6.57 | 2 | 164 | 5387 |
| **P1B** | endolith | 0.92 | 31.82 | 303 | 7.62 | 1 | 26 | 11372 |
| **PK03.19Kh** | endolith | 0.96 | 44.54 | 513 | 8.68 | 7 | 986 | 19024 |
| **P17Kh** | endolith | 0.94 | 31.12 | 250 | 7.47 | 1 | 32 | 8312 |
| **QKKRKh** | endolith | 0.92 | 21.59 | 184 | 6.94 | 4 | 364 | 6137 |
| **QRKRHh** | endolith | 0.92 | 39.56 | 278 | 7.48 | 17 | 882 | 10272 |
| **L21R** | endolith | 0.94 | 39.61 | 316 | 7.81 | 12 | 1012 | 14378 |
| **L22R** | endolith | 0.95 | 27.27 | 237 | 7.46 | 6 | 480 | 9458 |
| **L23R** | endolith | 0.91 | 23.73 | 180 | 6.82 | 7 | 378 | 10088 |
| **L28R** | endolith | 0.95 | 22.33 | 205 | 7.28 | 5 | 501 | 7156 |
| **L34R** | endolith | 0.93 | 21.68 | 190 | 7.02 | 4 | 57 | 6238 |
| **G29R** | endolith | 0.81 | 11.80 | 62 | 4.81 | 3 | 102 | 8151 |
| **ClK04.35R** | endolith | 0.96 | 39.20 | 236 | 7.55 | 3 | 303 | 8081 |
| **Cl1RdR** | endolith | 0.91 | 13.21 | 120 | 6.30 | 1 | 106 | 3797 |
| **ClD1R** | endolith | 0.93 | 18.37 | 190 | 7.00 | 1 | 38 | 6951 |
| **ClDRR** | endolith | 0.92 | 41.27 | 300 | 7.59 | 19 | 1252 | 11652 |
| **Cl32RR** | endolith | 0.94 | 38.35 | 313 | 7.76 | 17 | 1253 | 12602 |
| **C37R** | endolith | 0.95 | 25.50 | 248 | 7.54 | 7 | 325 | 10656 |
| **C38R** | endolith | 0.91 | 22.78 | 150 | 6.57 | 3 | 232 | 4896 |
| **C39R** | endolith | 0.93 | 19.24 | 117 | 6.42 | 4 | 145 | 5165 |
| **Q50K** | endolith | 0.92 | 34.44 | 230 | 7.22 | 8 | 628 | 8208 |
| **QK06.50bK** | endolith | 0.96 | 48.41 | 344 | 8.07 | 3 | 143 | 5799 |
| **Q45K** | endolith | 0.90 | 17.87 | 100 | 5.97 | 3 | 166 |  |
| **Q49bK** | endolith | 0.92 | 39.15 | 273 | 7.46 | 10 | 754 | 12810 |
| **QC2K** | endolith | 0.89 | 13.81 | 71 | 5.48 | 3 | 834 | 9257 |
| **QE014K** | endolith | 0.94 | 36.37 | 207 | 7.25 | 2 | 223 | 11468 |
| **GC1K** | endolith | 0.83 | 16.77 | 103 | 5.57 | 4 | 1498 | 5195 |
| **G53K** | endolith | 0.93 | 36.80 | 275 | 7.57 | 14 | 979 | 2822 |
| **G54RK** | endolith | 0.92 | 35.34 | 248 | 7.33 | 9 | 680 | 4705 |
| **A48bK** | endolith | 0.96 | 47.05 | 453 | 8.43 | 17 | 1382 | 22373 |
| **AK05.48K** | endolith | 0.96 | 34.19 | 244 | 7.62 | 9 | 384 | 8596 |
| **D46K** | endolith | 0.94 | 15.98 | 137 | 6.67 | 1 | 171 | 12224 |
| **R52K** | endolith | 0.94 | 20.82 | 144 | 6.71 | 6 | 387 |  |
| **K44K** | endolith | 0.95 | 45.40 | 433 | 8.30 | 16 | 1117 | 19373 |
| **B01** | BSC | 0.95 | 45.53 | 403 | 8.24 | 10 | 1087 | 17662 |
| **F01** | BSC | 0.95 | 46.06 | 551 | 8.68 | 6 | 328 | 23964 |
| **F02** | BSC | 0.92 | 32.97 | 301 | 7.56 | 4 | 2854 | 14644 |
| **F03** | BSC | 0.96 | 48.02 | 470 | 8.52 | 16 | 2821 | 20570 |
| **F04** | BSC | 0.95 | 42.08 | 484 | 8.47 | 8 | 1518 | 19523 |
| **F05** | BSC | 0.84 | 28.16 | 285 | 6.86 | 2 | 7864 | 19548 |
| **F06** | BSC | 0.96 | 49.49 | 543 | 8.71 | 5 | 1342 | 24777 |
| **F07** | BSC | 0.91 | 25.97 | 167 | 6.75 | 2 | 1654 | 6721 |
| **F08** | BSC | 0.95 | 47.99 | 413 | 8.27 | 18 | 1080 | 15733 |
| **F09** | BSC | 0.95 | 29.48 | 218 | 7.38 | 10 | 1561 | 9218 |
| **F10** | BSC | 0.95 | 33.50 | 340 | 8.01 | 12 | 746 | 14745 |
| **F11** | BSC | 0.92 | 34.72 | 246 | 7.30 | 9 | 769 | 10070 |
| **F12** | BSC | 0.95 | 46.13 | 402 | 8.19 | 16 | 1123 | 18361 |
| **F13** | BSC | 0.94 | 46.37 | 404 | 8.15 | 16 | 933 | 17147 |
| **F14** | BSC | 0.96 | 56.39 | 523 | 8.66 | 11 | 1036 | 21223 |
| **F15** | BSC | 0.95 | 41.11 | 330 | 7.94 | 10 | 590 | 10973 |
| **F16** | BSC | 0.96 | 62.61 | 697 | 9.10 | 26 | 1787 | 33511 |
| **F17** | BSC | 0.95 | 47.96 | 493 | 8.50 | 17 | 1517 | 21731 |
| **F18** | BSC | 0.94 | 43.49 | 356 | 7.95 | 13 | 953 | 15751 |
| **F19** | BSC | 0.95 | 48.38 | 504 | 8.50 | 18 | 1752 | 23860 |
| **F20** | BSC | 0.91 | 33.81 | 214 | 7.01 | 12 | 753 | 7742 |
| **F21** | BSC | 0.95 | 50.59 | 504 | 8.56 | 17 | 1267 | 23191 |
| **F22** | BSC | 0.95 | 47.06 | 459 | 8.43 | 17 | 1127 | 18612 |
| **F23** | BSC | 0.95 | 28.64 | 230 | 7.47 | 10 | 770 | 15450 |
| **F24** | BSC | 0.94 | 39.24 | 342 | 7.94 | 10 | 1188 | 15634 |
| **F25** | BSC | 0.94 | 46.51 | 405 | 8.16 | 15 | 1073 | 17611 |
| **S013** | BSC | 0.96 | 49.44 | 650 | 8.96 | 4 | 89 | 22371 |

^1^C_type - community type, ^2^P_evenness - Pielou's evenness index, ^3^Faith_pd - Faith's phylogenetic diversity index, ^4^B_ASV – number of ASVs (observed features) obtained for Bacteria, ^5^Shannon - Shannon diversity index, ^6^Cb_ASV - number of ASVs obtained for Cyanobacteria, ^7^Cb reads - number of reads acquired for Cyanobacteria, ^8^B_reads - number of reads acquired for Bacteria.

**Table S4.** Comparative analysis of endolithic cyanobacterial communities based on culture-dependent and culture-independent approach.

| **Sample ID** | **Cultured cyanobacteria. LM^1^** | **V3-V4 NGS analysis^2^** |
| --- | --- | --- |
| **G2B** | *Nodosilinea* sp. | Nostocaceae, Oxyphotobacteria unknown family *(Leptolyngbya* sp*./Phormidium* CYN64*)* |
| **G3B** | *Nodosilinea* sp., *Leptolyngbya* sp. | Phormidiaceae *(Planktothrix)* |
| **G4bB** | *Leptolyngbya* sp.*, Nodosilinea* sp. | Oxyphotobacteria unknown family *(Leptolyngbya* sp.*)* |
| **G7RB** | *Leptolyngbya* sp.*, Phormidium* sp. | Cyanobacteriia, Cyanobacteriaceae, Geitlerinemaceae, Gloeocapsaceae, Microcystaceae, Nostocaceae, Oscillatoriaceae, Paraspirulinaceae, Phormidiaceae, Oxyphotobacteria_Unknown_Family, Nodosilineaceae. Phormidesmiaceae, Sericytochromatia |
| **GK02.10B** | *Microcoleus sp. Synechococcus sp.* | Cyanobacteriia, Chroococcidiopsaceae, Thermosynechococcaceae. |
| **G12bB** | *Nostoc sp. Nodosilinea sp.* | Chroococcidiopsaceae |
|  |  |  |
| **G20B** | *Chroococcidiopsis* sp., *Nostoc* sp. | Cyanobacteriia, Cyanobacteriales, Chroococcidiopsaceae, Nostocaceae. Phormidiaceae |
| **L4B** | *Nodosilinea* sp., *Chroococcidiopsis* sp. | Cyanobacteriales, Chroococcidiopsidaceae, Coleofasciculaceae, Cyanobacteriaceae, Nostocaceae, Nodosilineaceae, Phormidiaceae |
| **L14aB** | *Chroococcidiopsis* sp*./Aliterella sp.* | Chroococcidiopsaceae |
| **P1B** | *Chroococcidiopsis* sp. | Cyanobacteriia, Chroococcidiopsaceae |
| **PK03.19B** | *Nostoc* sp.*, Phormidium* sp. | Chroococcidiopsaceae. Phormidiaceae. Thermosynechococcaceae. Sericytochromatia |
| **P17Kh** | *Chroococcidiopsis* sp. | Cyanobacteriia, Phormidiaceae |
| **QKKRB** | *Chroococcidiopsis* sp. | Chroococcidiopsaceae. Thermosynechococcacea |
| **QRKRB** | *Chrococcidiopsis* sp*.* | Cyanobacteriales, Cyanobacteriaceae, Geitlerinemaceae. Nostocaceae. Oscillatoriaceae, Phormidiaceae, Leptolyngbyaceae, Oxyphotobacteria_Unknown_Family, Nodosilineaceae, Phormidesmiaceae, Sericytochromatia |
| **L21R** | *Chroococcidiopsis* sp. */Aliterella. Leptolyngbza sp..* | Microcystaceae, Nostocaceae, Oscillatoriaceae, Paraspirulinaceae, Phormidiaceae, Leptolyngbyaceae, Oxyphotobacteria_Unknown_Family, Nodosilineaceae, Phormidesmiaceae, Pseudanabaenacea, Sericytochromatia |
| **L22R** | *Chroococcidiopsis* sp. | Chroococcidiopsaceae, Thermosynechococcaceae |
| **L23R** | *Croococcidiopsis* sp.*, Leprolyngby sp.* | Chroococcidiopsaceae, Phormidiaceae, Leptolyngbyaceae, Limnotrichaceae, Pseudanabaenaceae |
| **L24R** | *Croococcidiopsis* sp.*, Leprolyngbya* sp. *Synechococcus sp.* | Chroococcidiopsaceae, Microcystaceae, Phormidiaceae, Leptolyngbyaceae, Limnotrichaceae, Pseudanabaenaceae |
| **L28R** | *Chrococcidiopsis* sp., *Desertifilum sp.* | Chroococcidiopsaceae, Thermosynechococcaceae |
| **L34R** | *Chroococcidiopsis* sp., *Phormidium autumnalis, Leptolyngbya* sp. | Cyanobacteriia, Cyanobacteriales, Chroococcidiopsaceae |
|  |  |  |
| **G29R** | *Chrococcidiopsis* sp. *Phormidium* sp. | Nostocaceae, Oxyphotobacteria_Unknown_Family, Thermosynechococcaceae |
| **ClK04.35R** | *Wilmottia* sp. | Coleofasciculaceae, Nostocaceae, Phormidiaceae |
| **C1RdR** | *Croococcidiopsis* sp. | Chroococcidiopsaceae |
| **ClD1R** | *Chrococcidiopsis* sp. | Cyanobacteriia, Chroococcidiopsaceae |
| **ClDRR** | *Chrococcidiopsis* sp. | Chroococcidiopsaceae, Nostocaceae, Oscillatoriaceae, Paraspirulinaceae, Phormidiaceae, Leptolyngbyaceae, Oxyphotobacteria_Unknown_Family, Nodosilineaceae, Phormidesmiaceae, Sericytochromatia |
| **Cl32RR** | *Nodularia* sp. | Chroococcidiopsaceae, Cyanobacteriaceae, Nostocaceae, Oscillatoriaceae. Paraspirulinaceae, Phormidiaceae, Leptolyngbyaceae, Oxyphotobacteria_Unknown_Family, Nodosilineaceae, Phormidesmiaceae |
| **C37R** | *Chroococcidiopsis* sp. | Cyanobacteriales, Chroococcidiopsaceae, Nostocaceae, Phormidiaceae. Cyanobacteriales_uncultured |
| **C38R** | *Leptolyngbya* sp*., Chroococcidiopsis* sp. | Nostocaceae, Limnotrichaceae, Pseudanabaenaceae |
| **C39R** | *Chroococcidiopsis sp.* | Chroococcidiopsaceae, Limnotrichaceae, Nodosilineaceae, Vampirivibrionia |
|  |  |  |
| **Q50K** | *Leptolyngbya* sp., *Chroococcidiopsis* sp*.* | Nostocaceae. Oscillatoriaceae. Paraspirulinaceae. Leptolyngbyaceae. Oxyphotobacteria_Unknown_Family. Nodosilineaceae |
|  |  |  |
| **QK06.50bK** | *Leptolyngbya* sp., *Chroococcidiopsis* sp. | Cyanobacteriales, Phormidiaceae, Thermosynechococcaceae |
| **Q45K** | *Chroococcidiopsis* sp. | Chroococcidiopsaceae, Oscillatoriaceae, Oxyphotobacteria_Unknown_Family |
| **QE014K** | *Chroococcidiopsis* sp. | Chroococcidiopsacea |
|  |  |  |
| **Q49bK** | *Synechococcus* sp.*, Chroococcidiopsis* sp. | Nostocaceae. Oscillatoriaceae. Leptolyngbyaceae. Oxyphotobacteria_Unknown_Family. Leptolyngbyaceae. Oxyphotobacteria_Unknown_Family. Nodosilineaceae. Phormidesmiaceae. Sericytochromatia |
| **QC2K** | *Chrococcidiopsis* sp. | Cyanobacteriia. Chroococcidiopsaceae. Thermosynechococcaceae |
| **A48bK** | *Phormidium* sp. | Cyanobacteriaceae, Nostocaceae, Oscillatoriaceae. Paraspirulinaceae, Phormidiaceae, Leptolyngbyaceae, Oxyphotobacteria_Unknown_Family, Nodosilineaceae |
| **AK05.48K** | *Phormidium* sp. | Chroococcidiopsaceae. Coleofasciculaceae. Nostocaceae. Phormidiaceae, Sericytochromatia |
| **GC1K** | *Croococcidiopsis* sp. | Phormidiaceae, Thermosynechococcaceae |
| **G53K** | *Synechocystis* sp., *Chroococcidiopsis* sp. | Microcystaceae. Nostocaceae. Oscillatoriaceae. Paraspirulinaceae. Phormidiaceae. Leptolyngbyaceae. Oxyphotobacteria_Unknown_Family. Nodosilineaceae. Phormidesmiaceae. Sericytochromatia |
| **G54RK** | *Croococcidiopsis* sp. | Nostocaceae. Oscillatoriaceae. Oxyphotobacteria_Unknown_Family. Nodosilineaceae, Phormidesmiaceae. Sericytochromatia |
| **D46K** | *Phormidum/Oscillatoria* | Oscillatoriaceae |
| **R52K** | *Chroococcidiopsis* sp., *Phormidum* sp. | Cyanobacteriales. Chroococcidiopsaceae. |
|  |  |  |
| **K44K** | *Leptolyngbya* sp. | Geitlerinemaceae, Nostocaceae, Oscillatoriaceae, Paraspirulinaceae, Leptolyngbyaceae, Limnotrichaceae, Oxyphotobacteria_Unknown_Family, Nodosilineaceae, Phormidesmiaceae, Sericytochromatia |

^1^LM – light microscopy. ^2^V3-V4 NGS analysis – the next-generation sequencing based on the analysis of V3-V4 hypervariable region of 16S rDNA.

**Table S5.** The description of analyzed cyanobacterial strains.

**Strain CPC1 (E119)** cf. *Gloeocapsospsis* sp. Pamir E119. Cells appeared solitary. arranged in pairs or formed larger colonies of up to 32 cells. The size of mature cells varied between 3.5-5.5 μm. Envelopes were colorless and up to 0.3 μm thick. not lamellated. The initial step of cell division was membrane invagination; cell division was irregular in multiple planes. After division. the daughter cells were closely situated to each other. The cells after division were symmetrical. Low electron-dense inclusions. which can be gas vesicles – aerotopes. were observed (Figure 6. E119). They were not observed by light microscopy. The thylakoids were arranged parallel to the cytoplasmic membrane. Based on the 16S rRNA gene phylogeny the strain was located in the Gloeocapsopsis clade (Figure 7).

**Strain CP24R (E121)** cf. *Chroococcidiopsis* sp. Pamir E121. Cells were observed using light microscopy. they were solitary, arranged in pairs or formed larger colonies up to 32 cells. The size of mature cells varied between 2.8-5.5 μm. The mucilaginous sheaths were thick. colorless or yellowish to brown. not lamellated. Cell division was irregular in multiple planes and the beocytes have been noted. The initial step of cell division was membrane invagination. The daughter cells were closely situated to each other. The cells after division were symmetrical. The thylakoid arrangement was parietal with spherical formations which were rarely observed (Figure 6). The 16S rRNA gene-based phylogenetic analysis of the strain clustered with *Chroococcidiopsis* sp. but this needs further confirmation (Figure 7).

**Strain CP49R (E124)** cf. *Gloeocapsospsis* sp. Pamir E124. The size of mature cells was 4.1±1.3 μm. The sheath was colorless and attached to the cells. Thylakoids were regularly distributed in the cell. parallel to the cell wall; daughter cells were of different sizes and were closely situated to each other. The cells showed inclusions with low electron density which could be aerotopes with a regular (symmetrical) arrangement that was parallel to the thylakoids. On the 16S rRNA phylogenetic tree. the sequence is situated in the *Gloeocapsopsis* clade. grouping together with the strain E119 (Figure 7).

**Strain CP28 (E122)** cf. *Wilmottia* sp. Pamir E122. Simple trichomes. cylindrical. blue-green. pale blue green. or yellowish only slightly constricted at the cross–walls. Cells were longer than they were wide (2.7 ± 0.4 μm and 3.4 ± 0.9 μm respectively). The terminal cells were conical or rounded. without calyptra. Carboxysomes and other granules concentrated in the center of the cells were observed (Figure 6). Thin sheaths were closely attached to the trichomes. The ultrastructure of these cells resembled *Pseudanabaenaceae*. The thylakoids were arranged in parallel to the cytoplasmic membrane. Phylogenetic analysis of the 16S rRNA gene revealed that the sequence clustered with *Wilmottia* sp. (Figure 7).
