# Supplementary material for: Connectivity of Edaphic and Endolithic Microbial Niches in Cold Mountain Desert of Eastern Pamir (Tajikistan)
Source: Biology (Basel). 2021 Apr 9;10(4):314. doi: 10.3390/biology10040314 (PMC8069199; doi:10.3390/biology10040314)
Supplement: Supplementary file 1 [file biology-10-00314-s001.zip › Supplementary Figures Biology-1160517.doc]

**Figure S1.** Studied area (The map generated using the Google Earth Pro). The study areas which are marked in blue belong to Bulunkul, Rangkul and Karakul subregions.

**Figure S2**. The rarefaction curve showing the depth of the sequencing of V3-V4 hypervariable region of the 16S rRNA gene. Samples with low numbers of reads were removed from the samples’ set.

**Figure** **S3.** The box plot shows the fluctuation of the mean grain size (MGS) and mean grain area (MGA) for the samples of endoliths, excluding the reference samples due to their limited numbers.

**Figure S4**. The alpha diversity metrics calculated for communities inhabiting different types of rock reflected various microhabitats. C_type - the type of the community, P_evenness - Pielou's evenness index, Faith_pd - Faith's phylogenetic diversity index, B_ASV – number of ASVs obtained for Bacteria, Shannon - Shannon diversity index, Cb_ASV - number of ASVs obtained for Cyanobacteria, B_reads - number of reads acquired for Bacteria. The standard deviations calculated for endolithic communities and BSC in the case of P_evenness were 0.04 and 0.025, for Faith_pd they were 12.3 and 9.06, for Shannon they were 0.95 and 0.62, for Cb_ASV they were 5.4 and 5.7, for B_reads - 114.3 and 133.9.

(A)

(B)

**Figure S5**. The correlation plots reflect the relationships between the alpha diversity metrics and selected environmental parameters. (A) Spearman's rank correlation coefficient performed for endolithic and (B) for BSCs’ communities. Only statistically significant correlations are shown in the correlation plots.
